# Effect of a Novel Brief Motivational Intervention for Alcohol-Intoxicated Young Adults in the Emergency Department: A Randomized Clinical Trial

**DOI:** 10.1001/jamanetworkopen.2022.37563

**Published:** 2022-10-21

**Authors:** Jacques Gaume, Nicolas Bertholet, Jim McCambridge, Molly Magill, Angéline Adam, Olivier Hugli, Jean-Bernard Daeppen

**Affiliations:** 1 Department of Psychiatry—Addiction Medicine, Lausanne University Hospital and University of Lausanne, Lausanne, Switzerland; 2 Department of Health Sciences, University of York, York, United Kingdom; 3 Center for Alcohol and Addiction Studies, Brown University School of Public Health, Providence, Rhode Island; 4 Emergency Department, Lausanne University Hospital and University of Lausanne, Lausanne, Switzerland

## Abstract

**Importance:**

Heavy drinking among young adults is a major public health concern. Brief motivational interventions in the emergency department have shown promising but inconsistent results.

**Objective:**

To test whether young adults receiving a newly developed brief motivational intervention reduce their number of heavy drinking days and alcohol-related problems over 1 year compared with participants receiving brief advice.

**Design, Setting, and Participants:**

This randomized clinical trial was conducted at an emergency department of a tertiary care university hospital in Lausanne, Switzerland. Recruitment ran from December 2016 to August 2019. Follow-up was conducted after 1, 3, 6, and 12 months. All adults aged 18 to 35 years presenting for any cause and presenting with alcohol intoxication were eligible (N = 2108); 1764 were excluded or refused participation. Follow-up rate was 79% at 12 months and 89% of participants provided follow-up data at least once and were included in the primary analyses. Statistical analysis was performed from September 2020 to January 2021.

**Interventions:**

The novel intervention was based on motivational interviewing and comprised in-person discussion in the emergency department and up to 3 booster telephone calls. The control group received brief advice.

**Main Outcomes and Measures:**

Primary outcomes were the number of heavy drinking days (at least 60 g of ethanol) over the previous month and the total score on the Short Inventory of Problems (0-45, higher scores indicating more problems) over the previous 3 months. Hypotheses tested were formulated before data collection.

**Results:**

There were 344 young adults included (median [IQR] age: 23 [20-28] years; 84 women [24.4%]). Among the 306 participants providing at least 1 follow-up point, a statistically significant time × group interaction was observed (β = −0.03; 95% CI, −0.05 to 0.00; *P* = .02), and simple slopes indicated an increase of heavy drinking days over time in the control (β = 0.04; 95% CI, 0.02 to 0.05; *P* < .001) but not in the intervention group (β = 0.01; 95% CI, −0.01 to 0.03; *P* = .24). There was no effect on the Short Inventory of Problems score (β = −0.01; 95% CI, −0.03 to 0.02; *P* = .71).

**Conclusions and Relevance:**

This randomized clinical trial found that a brief motivational intervention implemented in the emergency department provided beneficial effects on heavy drinking, which accounts for a substantial portion of mortality and disease burden among young adults.

**Trial Registration:**

ISRCTN registry: 13832949

## Key Points

**Question:**

Does a newly developed brief motivational intervention help patients aged 18 to 35 years presenting to the emergency department with alcohol intoxication reduce heavy drinking and alcohol-related problems more than brief advice?

**Findings:**

In this randomized clinical trial, brief motivational intervention maintained a statistically significant lower number of heavy drinking days over 1 year compared with brief advice. No effects on alcohol-related problems were found.

**Meaning:**

These findings suggest that a brief motivational intervention model implemented in the emergency department among intoxicated young adults can have a beneficial effect on heavy drinking, which is a major public health concern.

## Teaser

This randomized clinical trial examines whether young adults receiving a newly developed brief motivational intervention reduce their number of heavy drinking days and alcohol-related problems over 1 year compared with receiving brief advice in the emergency department (ED).

## Introduction

Heavy episodic drinking (ie, at least 60 g of pure alcohol in a single occasion) is associated with an increased risk of injuries, trauma, violence, risky sexual behaviors, and other negative health outcomes, especially among young adults.^1,2^ Emergency department (ED) admissions related to alcohol intoxication generate a large burden on EDs internationally,^3,4,5,6,7,8^ which has increased over the last decade, particularly among young adults.^4,9,10,11^ Moreover, alcohol intoxication is associated with high likelihood of ED readmission and poorer psychiatric, substance use, and social outcomes over time.^12,13,14,15^

Brief intervention (BI) is an efficacious preventive strategy for alcohol consumption and its consequences,^16,17^ and its use in primary care is recommended by the World Health Organization and the US Preventive Services Task Force.^18,19^ However, systematic reviews have found mixed results regarding the efficacy of BI conducted in the ED among young adults (eg, improvements in both intervention and control groups with only some significant between-group differences,^20^ few differences in favor of ED-based BIs and poor study quality precluding firm conclusions,^21^ small but significant effect size for alcohol use but not for alcohol-related problems).^22^ Also, current evidence is specific to systematic screening and BI (ie, screening all patients and providing BI to those with hazardous use), and there are numerous barriers to implementation of this model.^23,24^ Given the challenges of BI implementation, one pragmatic approach could be to initiate BI with individuals presenting with intoxication in the ED. Detection of unhealthy alcohol use based on clinical presentation leads to the identification of individuals with more severe alcohol use,^25^ more likely to benefit from alcohol treatment–informed BI, such as brief motivational intervention (MI) enhanced by motivational interviewing techniques.^26^ Motivational interviewing is a person-centered counseling approach with a behavioral focus on resolving ambivalence in the direction of change.^27^ It is an evidence-based treatment for adult alcohol problems, demonstrating equivalence in effectiveness to more intensive psychological treatments while showing greater cost-effectiveness.^28,29^ Young adults are particularly receptive to motivational methods, which include acceptance, and avoidance of argumentation and confrontation.^30^ To our knowledge, only 4 studies have tested brief MI among young adults presenting to the ED while intoxicated and produced contrasted findings.^31,32,33,34^

We conducted a randomized clinical trial (RCT) testing the effects of a novel brief MI model for young adults presenting to the ED with alcohol intoxication, compared with a minimal intervention (brief advice [BA]). This RCT was embedded within a larger research program, in which we developed the brief MI,^35^ tested its effects in the present trial, and will later evaluate the mechanisms of effects. Our hypothesis was that participants receiving brief MI would reduce their number of heavy drinking days (HDD) and alcohol-related problems more than those receiving BA.

## Methods

### Study Design and Inclusion/Exclusion Procedures

This study was a single center, 2-group, parallel randomized clinical trial. The study protocol (including statistical analysis plan) is available in Supplement 1. The study was approved by the Ethics Committee of Canton Vaud, Switzerland and registered in the ISRCTN registry. All questionnaire data were recorded on a secure electronic database (eCRF) independently managed by the Clinical Trial Unit of Lausanne University Hospital. This report followed the Consolidated Standards of Reporting Trials (CONSORT) reporting guideline for randomized studies.^36^

**Figure 1.**
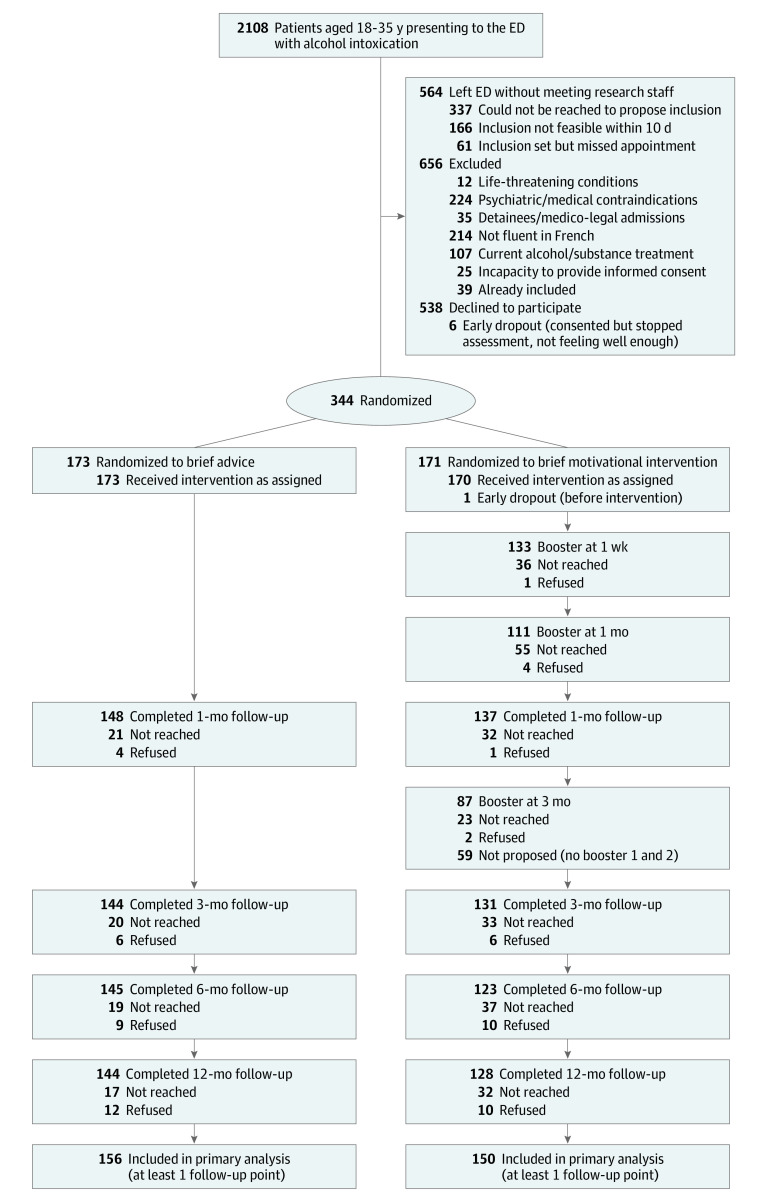
Trial Participation Diagram ED indicates emergency department.

Between December 2016 and August 2019, all patients aged 18 to 35 years presenting to Lausanne University Hospital ED for any cause and presenting with alcohol intoxication were eligible for study participation (Figure 1). Lausanne University Hospital serves as the primary care center for the city of Lausanne and surrounding borough, and as a tertiary care center for the region and neighboring states. Alcohol intoxication was assessed by ED staff based on either blood alcohol concentration (BAC) greater than or equal to 11.5 mmol/L (equivalent to 0.5 g/L), breathalyzer measure indicating BAC greater than or equal to 0.5 g/L, or clinical indication of intoxication as assessed by an ED physician. Based on earlier data,^4^ we recruited participants from 7AM to 12PM Thursday to Sunday initially, then Friday to Sunday from December 2017 onward. Patients meeting inclusion criterion but presenting outside of the investigators’ presence were contacted by phone and invited to come to Lausanne University Hospital Alcohol Treatment Center to participate in the study.

Patients were excluded if they had a life-threatening condition, psychiatric or medical contraindications, were detainees or presented for medico-legal reasons (ie, accompanied by the police), or did not speak French fluently (Figure 1). Research staff individually interviewed the remaining patients to further assess eligibility and excluded patients if they were currently receiving alcohol or substance use treatment. We used the University of California, San Diego Brief Assessment of Capacity to Consent^37^ to confirm patients capacity to consent and participate in research. Remaining patients provided written informed consent and a research clinician started their baseline assessment. Participants too unwell to complete the baseline assessment were excluded.

### Randomization and Blinding

After completing the computer-based assessment, the software automatically randomized the participant. Randomization sequence was generated without stratification, with a 1:1 allocation using random block sizes of 4 and 6. The algorithm was implemented by the Clinical Trial Unit independently of the field research staff and investigators. Participants were blinded to which kind of intervention they received. Both interventions were presented as “clinical interviews” and unblinding was provided at follow-up. Clinicians providing interventions could not be blinded.

### Follow-up

Participants were contacted for follow-up assessments at 1, 3, 6, and 12 months after baseline. These interviews were conducted by phone, using a computer-assisted program, by research assistants not involved in baseline procedures and blinded to patients’ group allocation and prior data. For all participants, incentives and reminders were used to compensate participation and enhance follow-up rates. Incentives were gift certificates of increasing value for each completed assessment (ie, 20 Swiss francs [CHF] for 1-month follow-up, 30 CHF for 3-month follow-up, 40 CHF for 6-month follow-up, and 50 CHF for 12-month follow-up [1 CHF is equal to approximately $1 US dollar]).

### Interventions

The brief MI model (Box) was developed and pretested in a previous phase of the project, described in details elsewhere.^35^ The control intervention (BA) included (1) brief structured feedback based on the Alcohol Use Disorders Identification Test (AUDIT) (ie, hazardous alcohol use based on a cut-off score of eight or more^38^, (2) information on alcohol risks based on a 1-page illustration leaflet, and (3) advice to reduce alcohol use and follow recommended guidelines provided on the reverse. Those with an AUDIT score greater than 16 (ie, probable alcohol use disorder) were offered referral to specialized alcohol treatment. The BA included no booster sessions.

Box. **Brief Motivational Intervention Description**

The brief MI used 3 strategies:
  Taking time to build a meaningful relationship by using relational factors such as empathy, acceptance, and collaboration.
  Eliciting patients’ change talk, softening their sustain talk, and strengthening their ability and commitment to change.
  Providing information and advice while supporting patients’ autonomy (including feedback and discussion about being admitted in the ED with alcohol intoxication).
The brief MI followed 3 steps:
  Exploring current situation (eg, ED admission context, emotional aspects, alcohol use) and important things in life (ie, raising ambivalence/discrepancy).
  Evoking change in a hypothetical future (ie, resolving ambivalence/decreasing discrepancy).
  Planning change (ie, concrete next steps).
When necessary, clinicians discussed and facilitated referral to alcohol treatment. After the baseline session, the clinician sent a letter summing up the discussion (ie, context, discussion, aims, and encouragements) to the participant by mail or email, according to participant’s choice.
Based on participant’s agreement, a booster session by phone was conducted after 1 week, 1 month, and 3 months to continue the discussion and follow-up participants’ progress and/or challenges.

Abbreviations: MI, motivational intervention; ED, emergency department.

Seven qualified research clinicians (master-level psychologists) provided both interventions, acting as addiction liaison consults for the study participants. ED staff did not deliver interventions in order to avoid additional workload and because liaison consults and interventions delivered by addiction specialists have been shown to have higher treatment fidelity.^39^ Research clinicians had clinical experience and basic motivational interviewing skills (ie, highly empathic and nonconfrontational style) that are important predictors of alcohol-related brief MI outcomes.^40,41^ Research clinicians were specifically trained to provide the interventions and supervision was provided biweekly throughout the project by a senior clinician expert in motivational interviewing.

### Primary and Secondary Outcomes

There were 2 primary outcomes. First, the number of HDD was derived from a 30-day timeline follow-back (TLFB) procedure.^42^ HDD was defined as a day with 6 standard drinks or more (ie, at least 60 g of ethanol).^19^ Second, alcohol-related problems were assessed using the 15-item Short Inventory of Problems (SIP) total score (version SIP-2R, possible range 0-45, higher scores indicating more problematic drinking).^43,44^ This measure has a 3-month timeframe and thus was assessed at 3-, 6-, and 12-month follow-ups.

Secondary outcome measures were: (1) weekly drinking amount derived from the TLFB at each follow-up time by summing the number of drinks each week and averaging it over the 4 weeks; (2) SIP sub-dimension scores (ie, physical, social, intrapersonal, interpersonal, and impulse control scores)^43,44^; (3) frequency of 4 additional alcohol-related consequences not covered by the SIP and developed specifically for young adults: unplanned sex, unprotected sex, and being a perpetrator or victim of violence,^45^ measured using the same scale as SIP (from 0 = never to 3 = every day or almost every day) at the same follow-up intervals; (4) proportions of participants with hazardous drinking at 12-month follow-up, based on AUDIT score of at least 8^38^ (the AUDIT has a timeframe of 12 months and was thus assessed only at this follow-up); (5) self-reported proportions of participants who started alcohol treatment, self-reported at 3-, 6-, and 12-month follow-up; (6) self-reported proportions of participants readmitted to the ED at 3-, 6-, and 12-month follow-up; (7) proportions of participants who started alcohol treatment; (8) proportions of participants who were readmitted to the ED according to Lausanne University Hospital medical records, consulted at 12-month follow-up (n = 325 [94.5%] granting consent); and (9) chronic heavy alcohol use based on ethyl glucuronide (EtG) concentration in head hair^46^ (eMethods in Supplement 2).

### Baseline Measures

Baseline measures included standard sociodemographic variables. Baseline alcohol use was measured using the AUDIT.^38^ We computed the proportion of participants with hazardous alcohol use (score of at least 8),^38^ as well as the AUDIT-C score^47^ which characterizes consumption patterns (first 3 items: frequency, quantity, and HDD). After question 3 of the AUDIT (ie, occurrence of HDD), we added a single item asking how often HDD happened over the last month to estimate baseline HDD. This method was preferred over a full TLFB at baseline to keep the questionnaire brief, first to minimize the impact of research procedures on clinical care, and second to reduce reactivity to alcohol assessment which has been shown as a source of bias.^48,49^ Finally, we used 2 visual analog scales with scores between 1 and 10 to measure importance of and confidence to change, as measures of baseline motivation to change alcohol use.^50,51,52^

### Intervention Fidelity

Intervention fidelity was measured using third-party observer ratings of audio-recorded sessions (Motivational Interviewing Skill Code).^53,54^ Detailed procedures are provided in eMethods and eTable 1 in Supplement 2.

### Sample Size Estimation

Using a program for power analysis in longitudinal design,^55^ a sample of 172 patients per group (with attrition of 20% over follow-ups) was required to detect small-medium effect sizes (0.25),^31,32,33,34,56^ with power set at 0.8, α at .05, and moderate autocorrelation dampening in generalized estimating equations for the primary outcomes.

### Statistical Analysis

Primary analyses used all available data in an intent-to-treat principle. We tested intervention effects over time by comparing groups on the primary and secondary outcomes. For repeated measures, analyses were conducted using generalized estimating equations (GEE).^57^ Because distribution was overdispersed, we used negative binomial distribution and log link for HDD, SIP scores, weekly drinking amount, and additional consequences. We used binomial distribution and logit link for self-reported ED readmission and initiation of alcohol treatment (ie, repeated dichotomous outcomes). All GEE models were set with an exchangeable correlation structure. For outcomes measured at 12-month follow-up only (ie, hazardous alcohol use, and ED readmission and starting alcohol treatment based on medical records), analyses were conducted using logistic regression models. Each model was adjusted for 1 baseline measure as follows: single item HDD for HDD outcome, AUDIT score for SIP scores, additional consequences, ED readmissions, and starting alcohol treatment, AUDIT-C score for weekly drinking amount, and baseline hazardous drinking for this same measure at 12-month follow-up. Chronic heavy use based on hair EtG was not baseline-adjusted but modeled across time (baseline, 6-, and 12-month follow-up). Significance threshold was set at *P* < .05.

Sensitivity analyses were conducted by repeating the aforementioned models (1) while adjusting for age and sex, (2) with robust standard error estimates, and (3) with multiple imputation of missing data.^58^ Attrition analyses tested whether baseline variables were associated with loss to follow-up using a GEE model with binomial distribution, logit link, and exchangeable correlation structure. Multivariate imputation using chained equations was computed in Stata BE version 17.0 (StataCorp), with 10 imputations, and distributions similar to those described previously. We then repeated primary analyses using the generated full data. Statistical analysis was performed from September 2020 to January 2021.

## Results

A total of 2108 young adults were eligible, 1544 were approached, and 344 were included (median [IQR] age, 23 [20-28] years; 84 women [24.4%]) (Figure 1). Other baseline descriptive statistics are presented in Table 1. Follow-up rate was 79% (272 of 344) at 12 months. Primary analyses using GEE included 306 participants (89%) who provided at least 1 follow-up point.

**Table 1.** Baseline Data

Characteristic	Participants, No. (%)		
	All (n = 344)	BA (n = 173)	Brief MI (n = 171)
Age, median (IQR), y	23 (20-28)	23 (20-28)	23 (20-27)
Sex			
Male	260 (75.6)	130 (75.1)	130 (76.0)
Female	84 (24.4)	43 (24.9)	41 (24.0)
Citizenship			
Swiss	226 (65.7)	111 (64.2)	115 (67.3)
Other^a^	118 (34.3)	62 (35.8)	56 (32.7)
Highest education level			
Obligatory school	94 (27.3)	51 (29.5)	43 (25.2)
Professional diploma	82 (23.8)	45 (26.0)	37 (21.6)
High school diploma	102 (29.7)	43 (24.9)	59 (34.5)
University degree	66 (19.2)	34 (19.7)	32 (18.7)
Heavy drinking days per month, median (IQR)^b^	2 (1-4)	1 (1-4)	2 (1-4)
AUDIT score, median (IQR)	13 (9-18)	12 (8-18)	13 (10-18)
AUDIT-C, median (IQR)	6 (4-8)	6 (4-8)	6 (5-8)
Importance to change, median (IQR)	5 (3-8)	5 (2-8)	5 (3-8)
Confidence to change, median (IQR)	8 (7-10)	8 (7-10)	8 (7-10)
Baseline hair EtG categories^c^			
No chronic heavy use (≤30 pg/mg)	81 (65.3)	39 (60.9)	42 (70.0)
Chronic heavy use (>30 pg/mg)	43 (34.7)	25 (39.1)	18 (30.0)

Abbreviations: AUDIT, Alcohol Use Disorder Identification Test; AUDIT-C, AUDIT Consumption score (first 3 items); BA, brief advice; EtG, ethyl-glucuronide; MI, motivational intervention.

^a^ For the list of countries included in other citizenships, see eTable 3 in Supplement 2.

^b^ Heavy drinking days using single item measure (see Methods).

^c^ Measured within a subsample having provided hair sample, N = 124 (36.2%), see eMethods in Supplement 2.

Intervention fidelity is presented in eTable 1 in Supplement 2. Tested measures consistently showed high fidelity.

**Figure 2.**
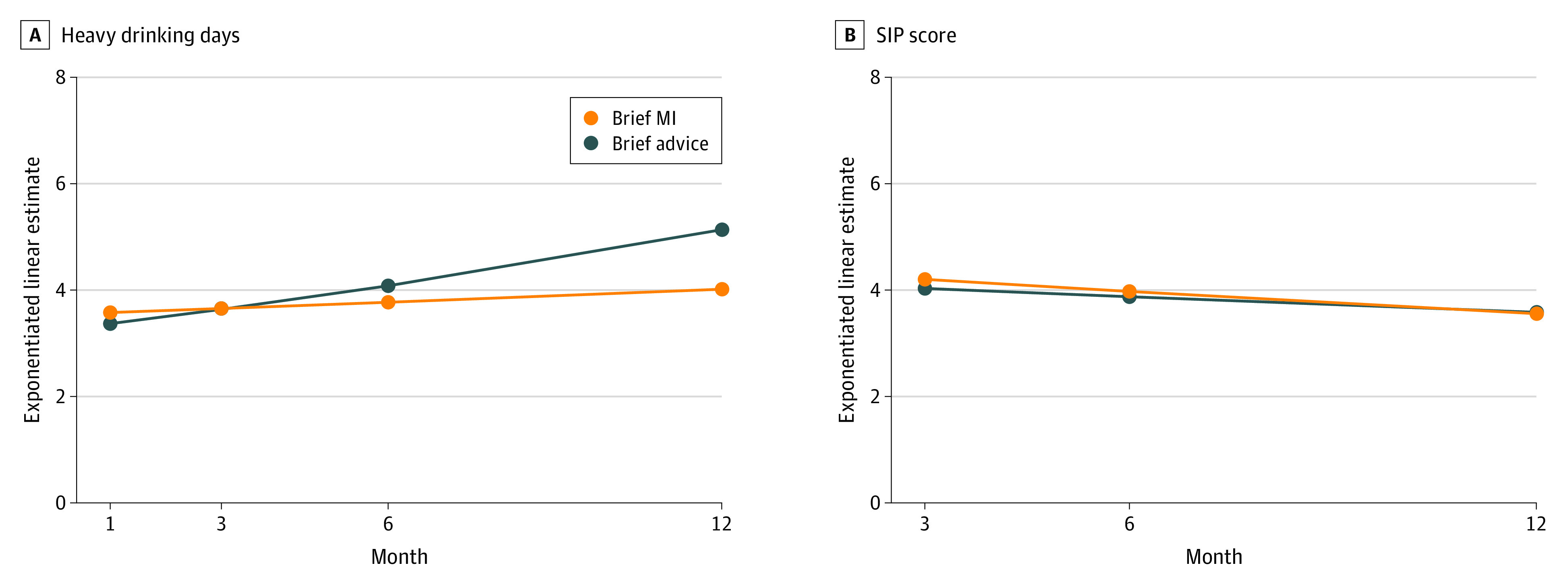
Primary Outcomes Over Follow-up by Intervention Groups MI indicates motivational intervention; SIP, Short Inventory of Problems.

The intervention effects are presented in Table 2, and Figure 2 depicts the effects on the primary outcomes, using interaction plots with marginal estimated values. We observed a significant time × intervention interaction (β = −0.03; 95% CI, −0.05 to 0.00; *P* = .02). The effect of time indicated a significant increase of HDD in the control group (β = 0.04; 95% CI, 0.02 to 0.05; *P* < .001), although this effect was not significant in the intervention group (β = 0.01; 95% CI, −0.01 to 0.03; *P* = 0.24). Based on marginal exponentiated linear estimated values over the follow-up time, the increase was of 0.4 HDD per month in the brief MI group vs an increase of 1.8 HDD per month in the control group. There were no differences in the other primary outcome (SIP score) (β = −0.01; 95% CI, −0.03 to 0.02; *P* = .71). In the secondary outcomes, only the hospital record of alcohol treatment initiation was significantly more likely in the brief MI group (Table 2).

**Table 2.** Intervention Effects

	Regression coefficient (SE) [95% CI]	*P* value
Heavy drinking days^a^		
Brief MI (vs BA)	0.09 (0.11) [−0.13 to 0.31]	.43
Time, mo^b^	0.04 (0.01) [0.02 to 0.05]	<.001
Brief MI × time	−0.03 (0.01) [−0.05 to 0.00]	.02
Short Inventory of Problems^a^		
Brief MI (vs BA)	0.06 (0.12) [−0.17 to 0.29]	.63
Time, mo^b^	−0.01 (0.01) [−0.03 to 0.01]	.18
Brief MI × time	−0.01 (0.01) [−0.03 to 0.02]	.71
Weekly drinking amount^a^		
Brief MI (vs BA)	0.09 (0.09) [−0.09 to 0.27]	.34
Time, mo^b^	0.03 (0.01) [0.01 to 0.04]	.002
Brief MI × time	−0.01 (0.01) [−0.03 to 0.01]	.37
Consequences^a^		
Brief MI (vs BA)	0.01 (0.17) [−0.32 to 0.34]	.95
Time, mo^b^	−0.02 (0.02) [−0.06 to 0.02]	.29
Brief MI × time	0.03 (0.03) [−0.02 to 0.09]	.24
Hazardous alcohol use^c^		
Brief MI (vs BA)	0.12 (0.27) [−0.41 to 0.65]	.67
Readmission in the ED (self-reported)^d^		
Brief MI (vs BA)	0.2 (0.29) [−0.37 to 0.77]	.50
Time, mo^b^	0.05 (0.04) [−0.03 to 0.12]	.24
Brief MI × time	−0.08 (0.06) [−0.19 to 0.03]	.15
Started alcohol treatment (self-reported)^d^		
Brief MI (vs BA)	0.85 (0.45) [−0.03 to 1.73]	.06
Time, mo^b^	0.03 (0.07) [−0.11 to 0.18]	.64
Brief MI × time	−0.11 (0.1) [−0.29 to 0.08]	.26
Readmission in the ED (medical record)^c^		
Brief MI (vs BA)	0.29 (0.33) [−0.35 to 0.93]	.37
Started alcohol treatment (medical record)^c^		
Brief MI (vs BA)	1.24 (0.58) [0.1 to 2.39]	.03
EtG indicating heavy use^e^		
Brief MI (vs BA)	−0.33 (0.29) [−0.89 to 0.23]	.25
Time (months)^b^	−0.06 (0.03) [−0.12 to 0]	.04
Brief MI × time	0.02 (0.04) [−0.07 to 0.1]	.68

Abbreviations: BA, brief advice; ED, emergency department; EtG, ethyl glucuronide; MI, motivational intervention.

^a^ Generalized estimating equation model with negative binomial distribution, log link, and exchangeable correlation structure; adjusted for a corresponding baseline measure (see Methods).

^b^ Follow-up months were mean-centered.

^c^ Logistic regression model; adjusted for a corresponding baseline measure (see Methods).

^d^ Generalized estimating equation model with binomial distribution, logit link, and exchangeable correlation structure; adjusted for a corresponding baseline measure (see Methods).

^e^ Generalized estimating equation model with binomial distribution, logit link, and exchangeable correlation structure.

Sensitivity analyses adjusting for age and sex and using robust standard error estimates supported all findings with similar patterns of significance and effect size (eTable 2 in Supplement 2). In attrition analyses, Swiss citizens (β = −0.49; 95% CI, −0.95 to −0.02; *P* = .04) and patients with university degrees (β = −0.98; 95% CI, −1.78 to −0.18; *P* = .02) were less likely to be lost to follow-up. Sex, age, and alcohol-related variables had no significant effects on attrition. Multiple imputation for missing data supported all findings with similar patterns of significance and effect size (eTable 2 in Supplement 2).

## Discussion

This randomized clinical trial found evidence that a novel ED-based brief MI model helped young adults with alcohol intoxication to maintain a lower level of HDD over 1 year, without having any effect on alcohol-related problems. Specifically, there was a significant increase of 1.8 HDD per month in the control group, while there was no significant increase in the brief MI group (+ 0.4 HDD per month over the follow-up time). This increasing HDD in the control group suggests that presenting to the ED while intoxicated is an opportunity for effective intervention (“teachable moment”).^59,60^ If missed, increased consumption over time may result in increased harm and associated health care costs. The beneficial effect of brief MI is in line with other trials in the ED showing positive alcohol use outcomes,^61^ including studies using brief MI with young adults.^22,32,56,62^ However, other studies have not found significant effects on alcohol use,^61^ and prominent among these are studies with young adults presenting to the ED while intoxicated.^31,33,34^ The latter studies targeted younger individuals (aged 13 to 17 years^33^ and 18 to 19 years^31^) or used shorter structured interventions (20-minute BI^34^) than the current study. It has been proposed that smaller effect sizes for BI and motivational interviewing among adolescents might be related to lower ambivalence to be resolved.^63^ Targeting older young adults and exploring and resolving ambivalence using longer motivational interviewing sessions might be an especially promising approach for future implementation.

While the beneficial effect of brief MI on HDD was significant, effects on other alcohol use measures and alcohol-related problems and consequences were absent. This is also in line with prior trials.^31,32,33,56^ Young adults in the brief MI group might not have decreased their alcohol use overall (weekly drinking amount) but might have changed their drinking pattern to avoid or limit intoxication (HDD). Alternatively, the observed changes in HDD might not have been sufficient to affect related consequences, would have required more time to have this effect, or were not captured by the measures used.^64^

Our brief MI model sought to encourage participants into specialized treatment when there were signs of severe alcohol problems. As expected with young adults, there were few referrals overall, but 13 of the 17 participants who initiated treatment were in the brief MI group, which translated to almost 4 times greater likelihood for brief MI participants compared with BA. Moreover, this is despite BA clinicians giving advice to consult with specialized treatment and providing an information leaflet with contact information for those whose AUDIT score was above 16. These results are important, since referral to treatment is a core feature of many BI models^65^ and provide evidence that may fill the knowledge gap regarding the benefits of interventions on the receipt of alcohol-related services, as highlighted by recent meta-analyses.^66,67^ However, they should be replicated before our new brief MI can be applied in other settings.

This study has several strengths. First, the brief MI model was carefully developed and pretested.^35^ Second, we deliberately chose a credible competitor as the control condition, in which MI features were absent. Third, we achieved high follow-up rates, and sensitivity analyses confirmed the pattern of results. Fourth, both interventions were delivered by clinicians carefully trained and supervised, resulting in high treatment fidelity. In addition, this trial incorporates a pragmatic implementation of our brief MI model, with intervention being delivered on a day-to-day basis by specialized addiction liaison clinicians, in collaboration with ED staff. Fifth, study outcomes were based on empirically supported self-report measures and objective measures such as biological outcome (hair EtG) and hospital records (ED readmission and/or alcohol treatment initiation).

### Limitations

Our study has several limitations. First, hair EtG could be collected in only one-third of participants, which resulted in reduced power to detect effects. Second, the hospital electronic medical records were accessed for 94.5% of the participants, but we might have missed ED visits and alcohol treatment in other hospitals or treatment facilities. Third, since we did not measure HDD, SIP, or most secondary outcomes at baseline as a consequence of the pragmatic nature of the study, we were not able to describe the progression of HDD and other outcomes from baseline to follow-up. Fourth, the brief MI condition included an initial session and up to 3 booster sessions, whereas the BA condition included a single session. We cannot rule out that the benefits seen were not due to the brief MI itself, but simply to the repeated contacts over time. Finally, this study was conducted in a single site in a Swiss university hospital; further replications are warranted in order to generalize findings to other contexts.

## Conclusions

Number of HDD is a major concern and accounts for a substantial portion of mortality and disease burden.^1,2,19,68^ This trial found that our novel brief MI can be implemented in the complex and sometimes chaotic ED setting and resulted in the stabilization of HDD over 1 year compared with the control group, whose heavy drinking increased. Also, alcohol treatment initiation was significantly more likely in the brief MI group compared with the BA group. However, our intervention had no effects on alcohol-related problems and other secondary outcomes. As our ultimate goal is to improve the impact of brief MI through optimization of training and implementation, an important next phase will be to identify treatment effect mediators to better understand intervention mechanisms and moderators to identify patients’ subgroups particularly benefiting from the intervention.^69^ This will allow us to refine the intervention, better tailored to engage young adults into reconsidering their heavy drinking while in the ED with alcohol intoxication and afterwards.

## Notes

Supplement 1.
Trial Protocol

Supplement 2. **eMethods.** Supplemental Methods
eReferences
**eTable 1.** Intervention Fidelity **eTable 2.** Sensitivity Analyses **eTable 3.** Other Countries of Citizenship

Supplement 3.
Data Sharing Statement
