# Supplementary material for: Effect of a Novel Brief Motivational Intervention for Alcohol-Intoxicated Young Adults in the Emergency Department: A Randomized Clinical Trial
Source: JAMA Netw Open. 2022 Oct 21;5(10):e2237563. doi: 10.1001/jamanetworkopen.2022.37563 (PMC9587483; doi:10.1001/jamanetworkopen.2022.37563)
Supplement: Supplement 1. — Trial Protocol [file jamanetwopen-e2237563-s001.pdf]

Clinical Study Protocol

Project PREMMIER

A Process study and Randomized controlled trial examining the Efficacy and Mechanisms of Motivational interviewing for alcohol Intoxicated young adults admitted to the Emergency Room

A Process study and Randomized controlled trial examining the Efficacy and Mechanisms of Motivational interviewing for alcohol Intoxicated young adults admitted to the Emergency Room (PREMMIER)

Clinical Study Protocol

Study Type:	Other Clinical Trial
Study Categorisation:	Category A
Study Registration:	**ISRCTN 13832949**<br>**https://doi.org/10.1186/ISRCTN13832949**<br>***(Information added 30.11.2016, before inclusion of participants in the RCT)***
Study Identifier:	Project PREMMIER<br>(Grant 105319_163123, Swiss National Science Foundation)
Sponsor-Investigator	Dr. Jacques Gaume, PhD<br>Responsable de recherche<br>CHUV<br>Service d'alcoologie<br>Av. Beaumont 21bis<br>1011 Lausanne<br>+41 21 314 41 05<br>Jacques.Gaume@chuv.ch
Investigational Product:	Brief motivational intervention to reduce alcohol use and related problems (psychosocial secondary prevention intervention)
Protocol Version and Date:	Version 3, 24.10.2016<br>***(Version authorized by the* Commission cantonale d'éthique de la recherche sur l'être humain *[Ethics Committee of Canton Vaud], 01/11/2016, ref: 2016-01476)***

Signature Page(s)

Study number	-
Study Title	A Process study and Randomized controlled trial examining the Efficacy and Mechanisms of Motivational interviewing for alcohol Intoxicated young adults admitted to the Emergency Room

The Sponsor-Investigator has approved the protocol version 3 (dated 24/10/2016), and confirm hereby to conduct the study according to the protocol, current version of the World Medical Association Declaration of Helsinki, ICH-GCP guidelines or ISO 14155 norm if applicable and the local legally applicable requirements.

Sponsor-Investigator: Jacques GAUME

Lausanne, 24.10.2016

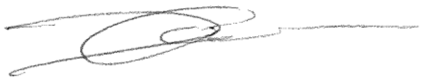

Place/Date Signature

TABLE OF CONTENTS

TABLE OF CONTENTS 4
STUDY SYNOPSIS 7
STUDY SUMMARY IN LOCAL LANGUAGE 13
ABBREVIATIONS 14
STUDY SCHEDULE 16
1. STUDY ADMINISTRATIVE STRUCTURE 17
1.1 Sponsor 17
1.2 Principal Investigator(s) 17
1.2.1 Principal Investigator 17
1.2.2 Co-Investigators 17
1.3 Statistician ("Biostatistician") 18
1.4 Laboratory 18
1.5 Monitoring institution 18
1.6 Data Safety Monitoring Committee 18
1.7 Any other relevant Committee, Person, Organisation, Institution 18
1.7.1 Data management and eCRF development 18
1.7.2 Clinical training and supervision for the motivational intervention 19
1.7.3 Supervision regarding clinical implementation of the study in the ER 19
1.7.4 Research staff 19
2. ETHICAL AND REGULATORY ASPECTS 21
2.1 Study registration 21
2.2 Categorization of study 21
2.3 Competent Ethics Committee (CEC) 21
2.4 Competent Authorities (CA) 21
2.5 Ethical Conduct of the Study 21
2.6 Declaration of interest 21
2.7 Patient Information and Informed Consent 21
2.8 Participant privacy and confidentiality 23
2.9 Early termination of the study 23
2.10 Protocol amendments 23
3. BACKGROUND AND RATIONALE 24
3.1 Background and Rationale 24
3.1.1 Alcohol use in young adults causes harm 24
3.1.2 Alcohol use and alcohol intoxication in the Emergency Room 24
3.1.3 Brief intervention and brief motivational intervention 24
3.1.4 BMI efficacy for young adults in the ER 24
3.1.5 Specific aims 25
3.2 Investigational treatment (prevention intervention) and Indication 25
3.3 Preclinical Evidence 25
3.4 Clinical Evidence to Date 25
3.5 Dose Rationale 27
3.6 Explanation for choice of comparator (or placebo) 27
3.7 Risks / Benefits 28
3.7.1 Anticipated risks. 28
3.7.2 Anticipated benefits and benefits/risks ratio. 28

3.8 Justification of choice of study population ... 28
4. STUDY OBJECTIVES ... 30
4.1 Overall Objective ... 30
4.2 Primary Objective ... 30
4.3 Secondary Objectives ... 30
4.4 Safety Objectives ... 30
5. STUDY OUTCOMES ... 31
5.1 Primary Outcome ... 31
5.2 Secondary Outcomes ... 31
5.3 Other Outcomes of Interest ... 32
5.4 Safety Outcomes ... 32
6. STUDY DESIGN ... 33
6.1 General study design and justification of design ... 33
6.1.1 Development and pre-test of a new intervention model (Aim 1) ... 33
6.1.2 Randomized controlled trial (Aim 2) ... 33
6.1.3 Mechanisms analysis (Aim 3) ... 34
6.2 Methods of minimising bias ... 35
6.2.1 Randomisation ... 35
6.2.2 Blinding procedures ... 35
6.2.3 Other methods of minimising bias ... 35
6.3 Unblinding Procedures (Code break) ... 35
7. STUDY POPULATION ... 36
7.1 Eligibility criteria ... 36
7.2 Recruitment and screening ... 36
7.3 Assignment to study groups ... 36
7.4 Criteria for withdrawal / discontinuation of participants ... 36
8. STUDY INTERVENTION ... 38
8.1 Identity of Investigational Products (treatment / medical device) ... 38
8.1.1 Experimental Intervention (treatment / medical device) ... 38
8.1.2 Control Intervention (standard/routine/comparator treatment / medical device) ... 38
8.1.3 Packaging, Labelling and Supply (re-supply) ... 38
8.1.4 Storage Conditions ... 38
8.2 Administration of experimental and control interventions ... 38
8.2.1 Experimental Intervention ... 38
8.2.2 Control Intervention ... 38
8.3 Dose / Device modifications ... 38
8.4 Compliance with study intervention ... 38
8.5 Data Collection and Follow-up for withdrawn participants ... 39
8.6 Trial specific preventive measures ... 39
8.7 Concomitant Interventions (treatments) ... 39
8.8 Study Drug / Medical Device Accountability ... 39
8.9 Return or Destruction of Study Drug / Medical Device ... 39
9. STUDY ASSESSMENTS ... 40
9.1 Study flow chart(s) / table of study procedures and assessments ... 40
9.2 Assessments of outcomes ... 41
9.2.1 Assessment of primary outcome ... 41
9.2.2 Assessment of secondary outcomes ... 41

9.2.3 Assessment of other outcomes of interest 42
9.2.4 Assessment of safety outcomes 42
9.2.5 Assessments in participants who prematurely stop the study 42
9.3 Procedures at each visit 42
9.3.1 Day 1 – Inclusion, Baseline procedures, Intervention (in the ER) 42
9.3.2 Month 1, by phone 43
9.3.3 Month 3, by phone 43
9.3.4 Month 6, by phone 43
9.3.5 Month 6, hair sampling (at Alcohol Treatment Centre) 44
9.3.6 Month 12, by phone 44
9.3.7 Month 12, hair sampling (at Alcohol Treatment Centre) 44
9.3.8 Month 12, not in presence of the participant 44
10. SAFETY 45
11. STATISTICAL METHODS 46
11.1 Hypothesis 46
11.2 Determination of Sample Size 46
11.3 Statistical criteria of termination of trial 46
11.4 Planned Analyses 46
11.4.1 Datasets to be analysed, analysis populations 46
11.4.2 Primary Analysis 47
11.4.3 Secondary Analyses 47
11.4.4 Interim analyses 48
11.4.5 Safety analysis 48
11.4.6 Deviation(s) from the original statistical plan 48
11.5 Handling of missing data and drop-outs 48
12. QUALITY ASSURANCE AND CONTROL 49
12.1 Data handling and record keeping / archiving 49
12.1.1 Case Report Forms 49
12.1.2 Specification of source documents 49
12.1.3 Record keeping / archiving 49
12.2 Data management 49
12.2.1 Data Management System 49
12.2.2 Data security, access and back-up 49
12.2.3 Analysis and archiving 50
12.2.4 Electronic and central data validation 50
12.3 Monitoring 50
12.4 Audits and Inspections 50
12.5 Confidentiality, Data Protection 50
12.6 Storage of biological material and related health data 51
13. PUBLICATION AND DISSEMINATION POLICY 52
14. FUNDING AND SUPPORT 53
14.1 Funding 53
14.2 Other Support 53
15. INSURANCE 53
16. REFERENCES 54
17. APPENDICES 59

STUDY SYNOPSIS

**Sponsor-Investigator**	Dr. Jacques Gaume, PhD
**Study Title:**	A Process study and Randomized controlled trial examining the Efficacy and Mechanisms of Motivational interviewing for alcohol Intoxicated young adults admitted to the Emergency Room
**Short Title / Study ID:**	Project PREMMIER
**Protocol Version and Date:**	Version 3, 24.10.2016
**Trial registration:**	**ISRCTN 13832949**<br>**https://doi.org/10.1186/ISRCTN13832949**<br>***(Information added 30.11.2016, before inclusion of participants in the RCT)***
**Study category and Rationale**	Other Clinical Trial, Category A.<br>The current clinical trial is neither a trial of therapeutic products or transplant products, nor a trial of transplantation.<br>The current clinical trial comes under Category A because:<br>• The health-related intervention investigated (i.e., a brief alcohol-related motivational interviewing) entails only minimal and temporary risks and burdens on participants' health (i.e., well-being).<br>• The health-related intervention investigated (i.e., a brief alcohol-related motivational interviewing) is recognized as standard in guidelines prepared in accordance with internationally accepted quality criteria.
**Clinical Phase:**	Not applicable
**Background and Rationale:**	Harmful alcohol use among young adults is a major public health concern and accounts for a significant portion of disease burden in Switzerland and worldwide (Rehm, 2011). In Switzerland, emergency room (ER) admissions for alcohol intoxication have increased substantially over the past decade, particularly among adolescents and young adults (Whiteford et al., 2013; Bertholet et al., 2014; Wicki & Stucki, 2014). Brief motivational interviewing (BMI) for young adults conducted in the ER have shown promising but inconsistent findings (Newton et al., 2013; Taggart, Ranney, Howland and Mello, 2013; Wicki et al., 2014). Recent studies on intervention mechanisms provide valuable insights on which mechanisms may be related to better outcomes (Gaume, McCambridge, Bertholet and Daeppen, 2014; Miller and Moyers, 2015). Built on past research, this study aims to develop and test a new BMI tailored to young adults admitted in the ER while intoxicated.

**Objective(s):**	Using a general mixed methods approach (e.g. Creswell, 2014), this project has the following 4 specific aims:<br>1. To develop a new motivational intervention model for young adults admitted in the ER with alcohol intoxication based on recent research on BMI mechanisms, and to pre-test it through qualitative methods.<br>2. To test the efficacy of this new model using a randomized controlled trial comparing it to a control condition receiving a minimal intervention (structured brief advice)<br>3. To evaluate the mechanisms of the intervention effects by evaluating moderators of the effect (i.e. under which circumstances the effect is observed), mediators of the effect (i.e. how the effect is translated into actual behavior change), moderated mediation (i.e. if the effect is transmitted through the designated mediators only under specific circumstances), as well as by exploring intervention successes and failures using qualitative analyses.<br>4. To finalize a validated version of the intervention model based on conclusions of the previous three aims and to disseminate it.
**Outcome(s):**	The primary outcomes will be<br>• number of heavy drinking days over the past 30 days<br>• alcohol-related problems over the past 30 days<br>Secondary outcomes will include<br>• Alcohol-related consequences<br>• Hazardous drinking status<br>• Starting alcohol treatment<br>• ER readmission.<br>In addition, biological outcome analysis (Ethyl glucuronide concentration in head hair) and administrative hospital data will confirm outcome measures, which classically rely on participants' self-report.
**Study design:**	The current study uses a mixed methods design (i.e. using both qualitative and quantitative analyses).<br>The major part of project is a two-arm parallel randomized controlled trial.

**Inclusion / Exclusion criteria:**	Participants (N = 356) will be young adults admitted in the ER with alcohol intoxication.<br>Inclusion criteria:<br>• Age between 18 and 35.<br>• Admitted in the ER (for any cause).<br>• Having alcohol intoxication (i.e., >0.5 gram/litter blood alcohol concentration or clinical indication of alcohol intoxication).<br>• Informed consent to participate in the trial<br>Exclusion criteria:<br>• Life-threatening conditions<br>• Detainees or medico-legal admissions.<br>• Not being fluent in French.<br>• Currently receiving another alcohol or substance use treatment.<br>• Psychiatric or medical contra-indications preventing patients understanding informed consent, fulfilling questionnaires, and participating in the intervention (evaluated using an adaptation of the University of California, San Diego Brief Assessment of Capacity to Consent; McCormack *et al.*, 2014).<br>Patients meeting inclusion criteria but being too intoxicated will be contacted by research staff once they have sobered up.

**Measurements and procedures:**	*Aim 1 – Intervention qualitative pre-tests*<br>Patients fulfilling the inclusion criteria will be contacted by a member of the research staff. After providing written informed consent, participants (N=12) will take part in an audio-recorded BMI session with a trained therapist. After the BMI, the participant will answer to a qualitative interview investigating his/her experience with the BMI.<br><br>*Aim 2 – Randomized controlled trial*<br>Inclusion and baseline assessment:<br>Patients fulfilling the inclusion criteria will be contacted by a member of the research staff. After providing written informed consent, participants (N=344) will be required to answer the baseline assessment questionnaires. The assessment (5-10 minutes) will be conducted in the ER using a tablet-based electronic questionnaire. After the assessment, the software will automatically randomize participants into the 2 groups: a) Brief advice (control condition), and b) BMI (experimental condition). All questionnaires and randomization will be conducted and directly recorded on a secured electronic case report form (eCRF, secuTrial) independently managed by the Clinical Trial Unit Lausanne.<br>Interventions:<br>Both brief advice (BA, control) and BMI (experimental) will be conducted while in the ER in a separate room providing confidentiality (please see descriptions below).<br>Follow-up:<br>Participants will complete a short post-session questionnaire while in the ER, and will then be contacted for follow-up assessments at 1, 3, 6, and 12 months post-baseline. Research assistants will conduct follow-up interviews by phone, using a computer assisted program directly recording data electronically in the eCRF (secuTrial). The interview will last 15 to 30 minutes.<br>Measures:<br>Primary outcome measures will be number of heavy drinking days and alcohol-related problems over the past 30 days, over the 4 follow-up times. Secondary outcome measures will be frequency of alcohol-related consequences, hazardous drinking status, alcohol treatment initiation, ER readmissions. Measures for intervention mechanisms study (Aim 3) will include readiness to change, self-efficacy, alcohol-related discrepancy and satisfaction with treatment. In addition, biological outcome analysis (Ethyl glucuronide concentration in head hair) and administrative hospital data will confirm outcome measures, which classically rely on participants' self-report. The latter measure will be part of an additional consent procedure.<br><br>*Aim 3 – Mechanisms analysis*<br>Participants will be asked for additional consent to audio-record intervention sessions. Then, audio-recorded sessions will be analysed using psycholinguistic coding.<br>In addition, a random sub-sample of 20 BA and 20 BMI sessions will be transcribed and analyzed using thematic analysis and recursive abstraction techniques (Maxwell, 2005). Furthermore, semi-structured interviews will be conducted after the 1-month and 12-month follow-up questionnaires with 10 patients randomly selected having received BA and 10 BMI sessions. Topics addressed will be qualitative assessment of potential short-/long-term changes experienced during the follow-up period and their relation or not with the intervention

	received.
**Study Product / Intervention:**	The BMI will be delivered while in the ER by a qualified research clinician (psychologist, clinical social worker, or nurse) and will last between 20 to 60 minutes. The intervention will use 3 main ingredients, in particular a) relational factors (e.g., empathy, acceptance, collaboration, avoidance of confrontation), b) evoking participants' change talk and strengthening their ability and commitment to change, and c) building a relationship significant in the long-run (e.g., including follow-up booster sessions). Overall, the intervention aims at increasing participants' motivation to change their drinking behaviours by enhancing discrepancy between their current behaviour and their broader life goals and values. The intervention focuses on helping participants to resolve the latter discrepancy through evoking and planning behaviour change. When necessary, interventionist will discuss and facilitate referral to alcohol treatment.<br>After the session, the interventionist will send a letter summing up the discussion (i.e., context, discussion, aims and encouragements) to the participant. Based on participant's agreement, booster session by phone will be conducted after **1 week** ~~**2 weeks**~~, 1 month, and 3 months.<br>***"2 weeks" changed to "1 week", 28.11.2016, (before inclusion of first participant)***
**Control Intervention (if applicable):**	Brief advice will be delivered in approximately 5 to 10 minutes. It will consist of a standardized tablet-based brief structured feedback and advice to cut down drinking with referral to specialist services for participants with alcohol use disorders.
**Number of Participants with Rationale:**	Power analysis were conducted and showed that a sample of N = 172 participants per group would be required to detect small/medium effect sizes (.25) in the planned analyses. The sample will thus include 344 participants. Primary analyses will use all data available from all participants included in the trial. Analysis will be performed in an intention to treat paradigm (i.e. participants having left the ER without finishing the intervention, and/or not receiving intervention booster sessions will be analyzed in the intervention group). In the event that we identify systematic mechanisms of missingness, we will explore varying techniques to impute missing data (Little and Rubin, 2002).<br>For Aim 1 and 3, samples were drawn to provide saturation of data, based on guidelines and on our experience in qualitative analysis.
**Study Duration and Study Schedule:**	The whole project is scheduled to be completed in 3 years.<br>Development and qualitative pre-tests (Aim 1) are scheduled over 3 months (August-October 2016).<br>RCT inclusion will last 18 months (December 2016-May 2018). Follow-up will be of 12 months for each participant and will thus finish in May 2019.

<table>
<tr><td>Investigator(s):</td><td>Jacques Gaume, principal investigator
CHUV
Service d’alcoologie
Av. Beaumont 21bis
1011 Lausanne
+41 21 314 41 05
Jacques.Gaume@chuv.ch

Jean-Bernard Daeppen, co-investigator
CHUV
Service d’alcoologie
Av. Beaumont 21bis
1011 Lausanne
+41 21 314 08 75
Jean-Bernard.Daeppen@chuv.ch

Nicolas Bertholet, co-investigator
CHUV
Service d’alcoologie
Av. Beaumont 21bis
1011 Lausanne
+41 21 314 73 51 (secretariat line)
Nicolas.Bertholet@chuv.ch

Oliver Hügli, co-investigator
CHUV
Services des urgences
1011 Lausanne
+41 21 314 05 67
Oliver.Hugli@chuv.ch

Prof. Molly Magill, international collaborator
Brown University
Center for Alcohol and Addiction Studies
Providence, Rhode Island 02912, USA
+1 (401) 863-6557
molly_magill@brown.edu

Prof. Jim McCambridge, international collaborator
University of York
Department of Health Sciences
Seebohm Rowntree Building
Heslington
York, Y010 5DD, UK
01904 32(1667)
jim.mccambridge@york.ac.uk</td></tr>
<tr><td>Study Centre(s):</td><td>Alcohol Treatment Centre, Lausanne University Hospital.</td></tr>
<tr><td>Statistical Considerations:</td><td>Intervention effects over time will be tested by comparing groups on the outcomes. For repeated measures, analyses will be conducted using generalized estimating equations with robust standard errors estimate.</td></tr>
<tr><td>GCP Statement:</td><td>This study will be conducted in compliance with the protocol, the current version of the Declaration of Helsinki, the ICH-GCP as well as all national legal and regulatory requirements.</td></tr>
</table>

STUDY SUMMARY IN LOCAL LANGUAGE

Développement et évaluation de l'efficacité d'un entretien motivationnel avec des jeunes adultes admis aux Urgences en ayant consommé de l'alcool

Contexte. La consommation d'alcool problématique au sein des jeunes adultes est fréquente et représente un problème de santé publique majeur. En effet, de nombreux jeunes adultes s'engagent régulièrement dans des épisodes de consommation d'alcool excessive (le fait de consommer 6 consommations d'alcool standards ou plus en une seule occasion), qui ont été associés à de nombreuses conséquences négatives, telles que des problèmes de santé divers, des accidents, des épisodes de violence interpersonnelle ou encore des comportements sexuels à risques. Ces comportements engendrent par ailleurs des coûts importants en matière de santé publique. La charge attribuée aux admissions liées à une consommation d'alcool problématique dans les services d'urgences hospitaliers est en effet considérable et a beaucoup augmenté parmi les jeunes adultes. Par exemple, le nombre de jeunes adultes admis au Service des urgences du CHUV avec une alcoolémie positive a quadruplé en une décennie. Ces constats nécessitent le développement de mesures de prévention adaptées. Parmi celles-ci, des interventions brèves motivationnelles visant la réduction de la consommation d'alcool problématique parmi les jeunes adultes ont été proposées. Bien que ce type d'interventions délivrées au sein des services d'urgences a montré des effets prometteurs, les résultats n'ont pas toujours été constants entre les études.

Objectifs: Cette étude vise à développer et à évaluer l'efficacité d'une nouvelle intervention brève motivationnelle délivrée aux urgences à des jeunes adultes admis avec une alcoolémie positive.

La première étape de ce projet vise à développer un nouveau modèle d’intervention motivationnelle, sur la base des connaissances scientifiques récentes et d’une démarche de recherche qualitative pour pré-tester ce nouveau modèle.

Pour tester l’efficacité de ce nouveau modèle, environ 350 jeunes adultes (18-35 ans) admis aux Urgences du CHUV avec une alcoolémie positive (>0.5 g/kg) seront ensuite inclus dans l'étude et attribués au hasard entre un groupe qui recevra la nouvelle intervention motivationnelle et un groupe qui recevra une intervention minimale. Tous les participants seront suivis pendant 1 an pour évaluer l’évolution de leur consommation d'alcool et des conséquences négatives associées.

Cette étude vise également à examiner les mécanismes de cette intervention afin de gagner une meilleure compréhension de ce qui la rend efficace ou pourrait la rendre plus efficace.

Méthodes : Un nouveau modèle d’intervention motivationnelle sera développé et pré-testé avec 12 participants au moyen de méthodes d’analyse qualitatives. Ensuite, environ 350 participants seront inclus dans l'étude et attribués au hasard entre un groupe qui recevra la nouvelle intervention brève motivationnelle (IBM) et un autre qui recevra une intervention brève classique (intervention brève de conseils structurés – IBCS – groupe contrôle). Tous les participants seront ensuite suivis pendant 1 an. En particulier, la consommation d'alcool et les conséquences négatives associées, l'initiation d'un traitement alcoologique et les réadmissions aux urgences seront évalués par téléphone 1, 3, 6 et 12 mois après l'inclusion dans l'étude. De plus, la permission de prélever un échantillon de cheveux sera demandée pour confirmer le niveau de consommation en analysant la concentration d’Ethyl-glucuronide et la permission d’accéder aux données administratives hospitalières sera demandée au participant pour confirmer l'initiation d'un traitement alcoologique et/ou les réadmissions aux urgences. Ces données permettront de comparer la nouvelles IBM et l'IBCS quant à leur efficacité respective à réduire la consommation d'alcool problématique. Pour examiner les mécanismes en œuvre lors de l'intervention, diverses mesures seront prises lors des évaluations de suivi, dont le sentiment d'auto-efficacité, la préparation au changement et la satisfaction par rapport à l'intervention.

Valeur attendue. La consommation problématique d'alcool parmi les jeunes représente un problème de santé publique majeur. La littérature indique que bien que les approches préventives délivrées aux urgences sont prometteuses, elles ont besoin d'être développées et renforcées. Répondant spécifiquement à ce besoin, cette étude est susceptible d'avoir un impact important en matière de santé publique.

ABBREVIATIONS

AE	Adverse Event
ASR	Annual Safety Report
ATC	Alcohol Treatment Center (Service d’alcoologie)
AUDIT score	Score at the Alcohol Use Disorder Identification Test
BA	Brief Advice
BAC	Blood alcohol concentration
BASEC	Business Administration System for Ethics Committees
BI	Brief Intervention
BMI	Brief Motivational Intervention
CA	Competent Authority (e.g. Swissmedic)
CEC	Competent Ethics Committee
CHUV	Centre hospitalier universitaire vaudois (Lausanne University Hospital)
CRF	Case Report Form
Co-I	Co-investigator
ClinO	Ordinance on Clinical Trials in Human Research *(in German: KlinV, in French: OClin)*
eCRF	Electronic Case Report Form
ER	Emergency Room
EtG	Ethyl-Glucuronide
GCP	Good Clinical Practice
GEE	Generalized estimating equations
IB	Investigator’s Brochure
Ho	Null hypothesis
H1	Alternative hypothesis
HDD	Heavy drinking days
ICH	International Council for Harmonisation of Technical Requirements for Pharmaceuticals for Human Use
IMP	Investigational Medicinal Product
IIT	Investigator-initiated Trial
ISF	Investigator Site File
ISO	International Organisation for Standardisation
ITT	Intention to treat
KOFAM	Koordinationstelle Forschung am Menschen / OFSP
LPTh	Loi sur les produits thérapeutiques
LRH	Loi fédérale relative à la recherche sur l’être humain
MD	Medical Device
MI	Motivational interviewing
MITI	Motivational interviewing treatment integrity
OClin	Ordonnance sur les essais cliniques dans le cadre de la recherche sur l'être

	humain *(in German : KlinV, in English : ClinO)*
PI	Principal Investigator
PIN	Personal identification number
RCT	Randomized controlled trial
SDV	Source Data Verification
SIP	Short Inventory of Problems
SNCTP	Swiss National Clinical Trials Portal
SOP	Standard Operating Procedure
SPC	Summary of product characteristics
SUSAR	Suspected Unexpected Serious Adverse Reaction
TLFB	Timeline Follow-back (alcohol use estimation technique)
TMF	Trial Master File

STUDY SCHEDULE

		2016					2017												2018												2019								
		Aug	Sep	Oct	Nov	Dec	Jan	Feb	Mar	Apr	May	Jun	Jul	Aug	Sep	Oct	Nov	Dec	Jan	Feb	Mar	Apr	May	Jun	Jul	Aug	Sep	Oct	Nov	Dec	Jan	Feb	Mar	Apr	May	Jun	Jul	Aug	Sep
		1	2	3	4	5	6	7	8	9	10	11	12	13	14	15	16	17	18	19	20	21	22	23	24	25	26	27	28	29	30	31	32	33	34	35	36	37	38
Phase 1	Experimental sessions 1	x																																					
	Experts consultation		x																																				
	Experimental session 2			x																																			
Phase 2	Clinical and research assistants' training				x																																		
	Programming (e.g., eCRF)	x	x	x																																			
	Patients inclusion					x	x	x	x	x	x	x	x	x	x	x	x	x	x	x	x	x	x																
	Interventions					x	x	x	x	x	x	x	x	x	x	x	x	x	x	x	x	x	x																
	Post-session questionnaire					x	x	x	x	x	x	x	x	x	x	x	x	x	x	x	x	x	x																
	Integrity coding and supervision					x	x	x	x	x	x	x	x	x	x	x	x	x	x	x	x	x	x																
	1-month follow-up						x	x	x	x	x	x	x	x	x	x	x	x	x	x	x	x	x	x															
	3-month follow-up								x	x	x	x	x	x	x	x	x	x	x	x	x	x	x	x	x	x													
	6-month follow-up											x	x	x	x	x	x	x	x	x	x	x	x	x	x	x	x	x	x										
	12-month follow-up																	x	x	x	x	x	x	x	x	x	x	x	x	x	x	x	x	x	x				
	Head hair/ ETG analysis																																			x	x		
	Statistical analyses - Baseline descrptives																							x	x	x	x												
	Statistical analyses - Efficacy																																			x	x	x	x
Phase 3	Session recording					x	x	x	x	x	x	x	x	x	x	x	x	x	x	x	x	x	x																
	Coding (psycho-linguistic)																				x	x	x	x	x	x	x	x	x	x	x								
	Coding data analyses																															x	x	x	x				
	Qualitative feedback 1-month						x	x	x	x	x	x	x	x	x	x	x	x	x	x	x	x	x	x															
	Qualitative feedback 12-month																	x	x	x	x	x	x	x	x	x	x	x	x	x	x	x	x	x	x				
	Qualitative data analyses - Interventions																						x	x	x	x													
	Qualitative data analyses - 1-month																							x	x	x	x												
	Qualitative data analyses - 12-month																																		x	x	x	x	
Phase 4	Model finalization and communication																																			x	x	x	x

1. STUDY ADMINISTRATIVE STRUCTURE

1.1 Sponsor

Centre Hospitalier Universitaire Vaudois (CHUV)
Rue du Bugnon 46, 1011 Lausanne
021 314 11 11

As the study sponsor and in accordance with legal provisions, Lausanne University Hospital will respond to any damages caused to participants.

1.2 Principal Investigator(s)

1.2.1 Principal Investigator

Dr Jacques Gaume, PhD
Responsable de recherche
CHUV
Service d'Alcoologie
Av. Beaumont 21bis
Bâtiment P2
1011 Lausanne
021 314 41 05
jacques.gaume@chuv.ch

Jacques Gaume will coordinate the whole study (i.e., data collection, analyses, scientific presentations in conferences and paper writing). He will also provide research staff with ongoing supervision and specialized training in motivational interviewing coding (i.e., motivational interviewing skill code; client language assessment-proximal/distal).

1.2.2 Co-Investigators

Prof. Jean-Bernard Daeppen
CHUV
Service d'Alcoologie
Av. Beaumont 21bis
Bâtiment P2
1011 Lausanne
021 314 08 75
jean-bernard.daeppen@chuv.ch

Dr Nicolas Bertholet, PD MER
CHUV
Service d'Alcoologie
Av. Beaumont 21bis
Bâtiment P2
1011 Lausanne
021 314 73 51
nicolas.bertholet@chuv.ch

Dr. Oliver Hügli, PD MER
CHUV
Service des Urgences
1011 Lausanne
021 314 05 67
olivier.hugli@chuv.ch

The three co-investigators will closely collaborate with the principal collaborator throughout the whole project by providing scientific and clinical expertise.

1.3 Statistician ("Biostatistician")

No statistician will be hired for this study. Main statistical analyses will be performed by PI, Dr. Jacques Gaume, who has a PhD and post-doctoral training in advanced quantitative analysis methods and has experience in analyzing data from 3 randomized controlled trials evaluating BMI (Daeppen *et al.*, 2011; Gaume *et al.*, 2011; Gaume *et al.*, *under review*). He will be assisted by Dr. Grazioli (see below Research collaborators).

In addition, the two international collaborators will provide advanced statistic methods consulting:

Prof. Molly Magill
Associate Research Professor and Director of Biostatistics
Brown University
Center for Alcohol and Addiction Studies
Providence, Rhode Island 02912, USA
+1 (401) 863-6557
molly_magill@brown.edu

Prof. Jim McCambridge
University of York
Department of Health Sciences
Seebohm Rowntree Building
York, Y010 5DD, UK
01904 32(1667)
jim.mccambridge@york.ac.uk

1.4 Laboratory

Forensic Toxicology and Chemistry Unit
Lausanne and Geneva Universities Centre of Legal Medicine
Ch. de la Vulliette 4
1000 Lausanne 25

This unit will be in charge of the head hair Ethyl glucuronide concentration analysis. In particular, Prof. Marc Augsburger (021 314 70 85, marc.augsburger@chuv.ch), will be in charge of this analysis and of the research assistants training to collect head hair samples.

1.5 Monitoring institution

Clinical Trial Unit Lausanne
Dr Marc Froissart, Dre Laure Vallotton
CHUV
Mont-Paisible 14
1011 Lausanne
021 314 61 84 / 021 314 90 59

The Clinical Trial Unit-Lausanne will be in charge of the study monitoring.

1.6 Data Safety Monitoring Committee

No Data Safety Monitoring Committee will be constituted for this study.

1.7 Any other relevant Committee, Person, Organisation, Institution

1.7.1 Data management and eCRF development

The Clinical Trial Unit-Lausanne, in particular M. Ali Maghraoui (ing) (021 314 42 65, ali.maghraoui@chuv.ch) will generate an electronic CRF (secuTrial software) to ease the data collection and to warrant the data integrity, security, quality and traceability (please see address above).

1.7.2 Clinical training and supervision for the motivational intervention

Cristiana Fortini
CHUV
Service d'Alcoologie
Av. Beaumont 21bis
Bâtiment P2
1011 Lausanne
021 314 46 71
cristiana.fortini@chuv.ch

Cristiana Fortini, senior psychologist and expert in motivational interviewing, will provide interventionists with training and ongoing supervision.

1.7.3 Supervision regarding clinical implementation of the study in the ER

In addition, integration of the present project with current clinical practice and patients flow in the ER will be carefully cared for throughout the whole project. The study PI will closely worked together with Co-Is Dr. Hugli, head physician at the ER, Dr. Daeppen, head physician at the ATC, Dr. Bertholet, associate physician at the ATC, Mrs. Fortini (see above) and the study clinicians to implement the study process and practices in the ER without interfering with the quality of the clinical practice, and to guarantee the ethical principle of treatment equity. Clinical supervision on these aspects will be ongoing throughout the project for study clinicians. Meeting of the clinical team, PI and Co-Is will be organized on a regular basis.

1.7.4 Research staff

1.7.4.1 Research collaborators

Sophie Paroz
Service d'Alcoologie
Av. Beaumont 21bis
Bâtiment P2
1011 Lausanne
021 314 73 51
sophie.paroz@chuv.ch

Sophie Paroz, social scientist and scientific collaborator at the Service d'alcoologie will provide expertise in qualitative methods.

Dr Véronique Grazioli, PhD
CHUV
Service d'Alcoologie
Av. Beaumont 21bis
Bâtiment P2
1011 Lausanne
021 314 73 51
veronique.grazioli@chuv.ch

Véronique Grazioli will work as a research collaborator over the 3-year study. Her main tasks will be to supervise the research assistants, to help the PI to coordinate the study (i.e., data collection, analyses, scientific presentations in conferences and paper writing), and conduct part of the qualitative data analysis.

1.7.4.2 Interventionists

Two or three interventionists will be hired over the study. They will conduct both types of interventions. They will also conduct patients' recruitment and inclusion, baseline assessment, and collect post-session patient's questionnaires.

1.7.4.3 Research assistants

Four research assistants will be hired over the study. Research assistants will be in charge of the follow-up assessments.

1.7.4.4 Psycholinguistic coders

Five coders will be hired over the study. Their tasks will encompass audio-recorded intervention sessions coding.

2. ETHICAL AND REGULATORY ASPECTS

2.1 Study registration

The current study will be registered in the ClinicalTrials.gov registry and in the supplementary federal database (Swiss National Clinical Trials Portal (SNCTP), through BASEC application.

Registered in the ISRCTN, # 13832949, https://doi.org/10.1186/ISRCTN13832949

(Information added 30.11.2016, before inclusion of participants in the RCT)

2.2 Categorization of study

The current clinical trial comes under ***Other clinical trial, category A*** because it is neither a trial of therapeutic products or transplants products, nor a trial of transplantation. Next, the health-related intervention investigated (i.e., a brief alcohol-related motivational interviewing) entails only minimal and temporary risks and burdens on participants' health (i.e., well-being). Finally, the health-related intervention investigated is recognized as standard in guidelines prepared in accordance with internationally accepted quality criteria.

2.3 Competent Ethics Committee (CEC)

The responsible investigator ensures that approval from an appropriately constituted Competent Ethics Committee (CEC) is sought for the clinical study.

No significant change will be made to the protocol without CEC approval, except where necessary to eliminate apparent immediate hazards to study participants. In case of a significant change, the principal investigator will submit to the CEC any application documents specified in Annex 3 of ClinO that are affected by the change, while providing information on the reasons for the change. Examples of significant changes in the current study may encompass changes affecting participants' rights and obligations, changes to the protocol (e.g., changes to eligibility criteria, method of investigation, endpoints, form of statistical analysis), conducting the clinical trial at an additional site, or a change of sponsor, coordinating investigator or investigator responsible. Other minor changes will be notified to the CEC in the annual safety report.

In case of premature study end or interruption of the study, it would be reported within 15 days to the CEC. Next, the regular end of the study will be reported within 90 days and the final study report will be submitted within one year after the study end.

2.4 Competent Authorities (CA)

Not applicable (category A).

2.5 Ethical Conduct of the Study

The study will be carried out in accordance to the protocol and with principles enunciated in the current version of the Declaration of Helsinki and the guidelines of Good Clinical Practices (GCP) issued by ICH. The CEC will receive annual safety report and interim reports and be informed about study stop/end in agreements with local requirements.

2.6 Declaration of interest

The principal investigator and the co-investigators declare that they have no conflict of interest.

2.7 Patient Information and Informed Consent

Informed consent process will be conducted prior to inclusion by trained and delegated research clinicians. Research clinicians will explain to each participant (meeting the inclusion criteria) the nature of the study and its aims, the procedures involved, the expected duration, the potential risks and benefits it may entail, and the financial compensation (gift certificates of increased value for

each follow-up assessment completed, i.e. 20, 30, 40, and 50 CHF for the 1-, 3-, 6-, and 12-month follow-up, respectively). Each participant will be informed that study participation is voluntary and he/she may withdraw from the study at any time and that study withdrawal will not affect his/her subsequent medical treatment.

As allowed by Article 18 of the Swiss Federal Act on Research involving Human Beings (Human Research Act, HRA), the information sheet will provide incomplete information regarding the randomization to different interventions. The information sheet will not mention the two distinct intervention groups to avoid a source of bias due to potentially different behavior depending of the intervention that participants will be randomized to. As required by Art. 18 HRA, this information will be provided, but at the end of the follow-up. At that time, the participant will have the possibility to consent to the use of the collected data or to refuse this use. In agreement with Art. 18 HRA, paragraph 3, data will be used only if the participant consents to it. All participants will receive a participant information sheet and a consent form (see Appendices) describing the study and providing sufficient information for participant to make an informed consent decision about their participation in the study. Research clinicians will be required to take time to ensure that all participants understand the nature of the study, its purposes, duration and the potential risks and benefits it entails. The latter points will therefore been carefully explained by trained research assistants. After providing all information, research clinicians will administrate the University of California, San Diego Brief Assessment of Capacity to Consent (UBACC; Jeste, 2007) to ensure that all participants understand study information. The UBACC was developped as a practical instrument to assess decision-making capacity. It has been successfully used to screen for capacity to consent within a research among frequent ER users with severe alcohol use disorders, which are often excluded from research, in part because assessing capacity to provide consent is challenging (McCormack et al., 2014). Participants will then been given time to read carefully the participation information sheet (which will cover the points discussed with the research clinicians) and the consent form. Enough time will given to the participants to decide if he/she wants to participate in the study. The research clinicians will answer all questions and provide participants with additional information when necessary.

The participants will be ask if they consent to participate in the questionnaires (baseline and follow-up) and the intervention which are the essential parts of the study. Additional (optional consent) will be asked for the audio-recording of the intervention and consultation of their hospital administrative and medical records. Participants refusing these optional consents will not be excluded from the study.

The participant will date and sign the informed consent form. Then the form will be dated and signed by the research clinician. A copy of the fully signed informed consent will be given to the participant together with the information sheet. The originally signed informed consent will be retained as part of the study records.

An additional consent procedure will be conducted at the end of the baseline procedures. Participants will be asked for consent to take a 2-3 centimeters long strand of hair (diameter of pencil lead) for testing of Ethyl glucuronide (EtG) at baseline, 6-, and 12-month follow-up. EtG in head hair has been presented as the only biomarker that can conclusively discriminate active heavy drinkers from social and non-drinkers (Crunelle *et al.*, 2014). EtG concentration will be a secondary, confirmatory outcome measure. Participants with head hair shorter than 2 centimeters or refusing hair sampling will not be excluded from the study. An additional consent procedure at the end of the baseline procedure since the sampling might be felt as more invasive than questionnaires and brief intervention and might lower participant willingness to participate to the essential parts of the study.

During the pre-testing phase (Aim 1), information and consent procedure will also be used (see Appendices). The participants will be ask if they consent to participate in an audio-recorded brief motivational intervention and a semi-structured qualitative interview. There will be no questionnaires and no follow-up.

For mechanisms analyses (Aim 3), 15-20 minute qualitative semi-structured interview are planned after follow-up questionnaire for a randomly selected sub-group (N=10 participants in BMI group and N=10 in BA group at 1-month follow-up; and N=10 participants in BMI group and N=10 in BA group at 12-month). This interview will happen right after follow-up questionnaire interview. At that moment, the interviewer will provide information on the type of questions, their goal, and the resulting increase in questionnaire length. As for any other follow-up questions, participants will be allowed not to answer these questions.

2.8 Participant privacy and confidentiality

A special attention will be given to maintain the confidentiality of data provided by the participants. Direct subject identifiers (e.g., participants' names, contact information) will be collected to facilitate follow-up communication and thereby conduct the whole study. To ensure confidentiality of data, all data collected on participants in the eCRF—including lab tests (i.e., head hair samples)—will be identified with a randomly generated, unique participant identification number (PIN). Master lists of PINs and individually identifiable private information will be stored in locked file cabinets and password-protected computers with restricted access. These lists will be available only to research staff on this project. The linkage and the direct subject identifiers will be destroyed 10 years after study completion.

For data verification purposes, authorized representatives of the Sponsor (the Clinical Trial Unit-Lausanne) or a CEC may require direct access to parts of the medical records relevant to the study, including participants' medical records.

Information regarding privacy and confidentiality will stand in the information sheet. It will also be specified that the principal investigator will permit trial-related monitoring, audits or CEC reviews, which may entail provision of direct access to participants' identifiers.

2.9 Early termination of the study

The Sponsor-investigator may terminate the study in the case of insufficient participant recruitment. That being said, based on past research conducted in similar settings in Lausanne (Daeppen et al., 2007) and elsewhere (Monti et al., 1999; Smith et al., 2003; Spirto et al., 2004), this scenario is relatively unlikely.

Due to the restricted level of risk for participants, no early termination of the study for safety reasons are anticipated (see Chapter 10).

2.10 Protocol amendments

Significant protocol modifications (defined in ClinO Art. 29) will be implemented after CEC approval only. Under emergency circumstances, deviations from the protocol to protect rights, safety and well-being of human subjects may proceed without prior approval of the CEC. Although very unlikely, if such deviations happen, it will be documented and reported to the CEC as soon as possible.

All non-significant changes are communicated to the CEC within the Annual Safety Report (ASR).

3. BACKGROUND AND RATIONALE

3.1 Background and Rationale

3.1.1 Alcohol use in young adults causes harm

Globally, harmful alcohol use results in approximately 2.5 million deaths annually and about 9% of these deaths are among young people between the ages of 15 and 29 (WHO, 2011). These deaths are mainly the result of alcohol-related motor vehicle accidents, homicides, suicides, and drowning. Heavy drinking episodes (i.e. drinking 6 standard drinks or more [>60 grams of pure alcohol] in a single occasion) and acute alcohol intoxication are associated with an increased risk of injuries, trauma, violence, risky sexual behaviors, and other negative health outcomes, especially among young adults (Rehm, 2011). Heavy drinking episodes during adolescence have also been related to an increased risk of alcohol dependence, other substances abuse, psychiatric comorbidities, and social difficulties in adulthood (McCambridge *et al.*, 2011).

3.1.2 Alcohol use and alcohol intoxication in the Emergency Room

The burden of unhealthy alcohol use on hospitals is noteworthy. Emergency room (ER) admissions related to alcohol intoxication represent a large burden on the ER clinical teams and account for a significant part of the resources in ERs (Pirmohamed *et al.*, 2000; Imlach *et al.*, 2011; Verelst *et al.*, 2012; Bertholet *et al.*, 2014). In Switzerland, ER admissions for alcohol intoxication have increased over the last decade, among all age groups (+11%), but particularly among adolescents and young adults (+57% among 10-23 years old; (Wicki and Stucki, 2014). At Lausanne University Hospital ER, the number of young adults between 18 and 30 years old admitted with a positive blood alcohol concentration (BAC ≥ 0.5 gram/liter) increased fourfold between 2000 and 2011 (from 125 to 544 cases). In 2011, 30% of all positive BACs recorded in this ER concerned young adults from 18-30 years old. Moreover, first results of a study on health care utilization by young adults admitted in this ER with alcohol intoxication showed that more than half of these patients were readmitted over the next 6 years, and 24% for a new alcohol intoxication episode.

3.1.3 Brief intervention and brief motivational intervention

While young adults accrue significant harm due to heavy alcohol use, studies of the natural history of alcohol use disorders have shown that the likelihood of such disorders is lower among younger individuals than it is among older individuals; .if present, they are probably milder in severity (e.g. Schuckit *et al.*, 1995). Therefore, secondary prevention interventions are likely to be of substantial benefit with younger individuals (Sommers *et al.*, 2013). Reviews on strategies targeting alcohol use show that brief interventions (BI) are among the few effective preventive strategies and the most cost-effective strategy among individual-centered approaches (Babor *et al.*, 2010). Structured brief advice is the most common BI and appears to lend itself to wide implementation, though it might not be adequate for addressing more severe alcohol problems (McCambridge and Rollnick, 2014). The other principal type of BI is brief motivational intervention (BMI), i.e. brief adaptations of motivational interviewing (MI). MI combines the person-centered counseling approach originally developed by Carl Rogers with a behavioral focus on resolving ambivalence in the direction of change (Miller and Rollnick, 2013). McCambridge and Rollnick (2014) have proposed that targeting alcohol problems directly with high quality MI as a BI is a promising route for further study. MI is an evidence-based treatment for adult alcohol problems, demonstrating equivalence in effectiveness to more intensive psychological treatments while showing greater cost effectiveness (e.g. Miller and Wilbourne, 2002; Lundahl and Burke, 2009). Adolescents and young adults are particularly receptive to motivational methods because they include acceptance, avoidance of argumentation and hostile confrontation, and eschew giving lectures or ultimatums (Tevyaw and Monti, 2004).

3.1.4 BMI efficacy for young adults in the ER

Two recent systematic reviews addressed the efficacy of BMI conducted in the ER for young adults and both found mixed findings (Newton *et al.*, 2013; Taggart *et al.*, 2013). Newton *et al.* (2013) also noted poor study quality precluding firm conclusions for many comparisons. A recent meta-analysis of alcohol BMIs for adolescents and young adults (Tanner-Smith and Lipsey, 2014), however, showed that BI led to significant reductions in alcohol consumption (effect size = 0.17) among young

adults 19 to 30 years old. Tests of intervention characteristics as potential moderators showed smaller but significant effect size in the ER settings (effect size = 0.11).

The studies summarized above typically used a screening process to include participants. Therefore, results might not be generalizable to populations admitted in the ER while intoxicated. One recent systematic review (Wicki *et al.*, 2014) investigated the efficacy of interventions among this specific population. The authors found 8 studies, including 4 studies comparing MI to standard care among young adults. Three of these showed results favoring MI. Monti *et al.* (1999) found significant differences on alcohol-related re-injuries and problems, and drinking and driving among 94 young adults (age 18-19). Smith *et al.* (2003) found significant differences on alcohol use and on alcohol-related problems among 151 young adults with facial injury (age 16-35). Spirito *et al.* (2004) found no significant effects among 152 adolescents (age 13-17) overall, but found significant effects on alcohol drinking days and heavy drinking episodes when limiting analyses to those reporting pre-existing problematic alcohol use. Only one study had null findings (Sommers *et al.*, 2006); it included an older sample (18 to 45) comprised of motor vehicle crash victims, excluded patients with higher alcohol problem severity, and evaluated a 15-20 minute MI as a complement to a 5-25 minute health interview.

Wicki et al. (2014) concluded that MI had a clear added value when compared to standard care, at least at short-term follow-up. However, they noted that it remained unclear which elements were related to efficacy. Recent research on intervention mechanisms has provided valuable insights on which mechanisms may be related to better outcomes (Gaume *et al.*, 2014; McCambridge and Rollnick, 2014; Miller and Moyers, 2015).

3.1.5 Specific aims

Using a general mixed methods approach (e.g. Creswell, 2014), this project has the following 4 specific aims:

1. To develop a new motivational intervention model for patients admitted in the ER with alcohol intoxication based on recent research on BI and MI mechanisms, and to pre-test it through qualitative methods.
2. To test the efficacy of this new model using a randomized controlled trial comparing it to a control condition receiving a minimal intervention (structured brief advice)
3. To evaluate the mechanisms of the intervention effects by evaluating moderators of the effect (i.e. under which circumstances the effect is observed), mediators of the effect (i.e. how the effect is translated into actual behavior change), moderated mediation (i.e. if the effect is transmitted through the designated mediators only under specific circumstances), as well as by exploring intervention successes and failures using qualitative analyses.
4. To finalize a validated version of the intervention model based on conclusions of the previous three aims and to disseminate it.

3.2 Investigational treatment (prevention intervention) and Indication

This study aims at developing and testing a new motivational intervention model for patients admitted in the ER with alcohol intoxication. This motivational intervention will be a brief adaptation of motivational interviewing (MI) (Miller and Rollnick, 2013). MI combines the person-centered counseling approach originally developed by Carl Rogers with a behavioral focus on resolving ambivalence in the direction of change (Miller and Rollnick, 2013).

3.3 Preclinical Evidence

Not applicable.

3.4 Clinical Evidence to Date

Two recent systematic reviews addressed the efficacy of BMI conducted in the ER for young adults and both found mixed findings (Newton *et al.*, 2013; Taggart *et al.*, 2013). Newton *et al.* (2013) also noted poor study quality precluding firm conclusions for many comparisons. A recent meta-analysis of alcohol BMIs for adolescents and young adults (Tanner-Smith and Lipsey, 2014), however,

showed that BI led to significant reductions in alcohol consumption (effect size = 0.17) among young adults 19 to 30 years old. Tests of intervention characteristics as potential moderators showed smaller but significant effect size in the ER settings (effect size = 0.11).

The studies summarized above typically used a screening process to include participants. Therefore, results might not be generalizable to populations admitted in the ER while intoxicated. One recent systematic review (Wicki *et al.*, 2014) investigated the efficacy of interventions among this specific population. The authors found 8 studies, including 4 studies comparing MI to standard care among young adults. Three of these showed results favoring MI. Monti *et al.* (1999) found significant differences on alcohol-related re-injuries and problems, and drinking and driving among 94 young adults (age 18-19). Smith *et al.* (2003) found significant differences on alcohol use and on alcohol-related problems among 151 young adults with facial injury (age 16-35). Spirito *et al.* (2004) found no significant effects among 152 adolescents (age 13-17) overall, but found significant effects on alcohol drinking days and heavy drinking episodes when limiting analyses to those reporting pre-existing problematic alcohol use. Only one study had null findings (Sommers *et al.*, 2006); it included an older sample (18 to 45) comprised of motor vehicle crash victims, excluded patients with higher alcohol problem severity, and evaluated a 15-20 minute MI as a complement to a 5-25 minute health interview.

Wicki et al. (2014) concluded that MI had a clear added value when compared to standard care, at least at short-term follow-up. However, they noted that it remained unclear which elements were related to efficacy. Recent research on intervention mechanisms has provided valuable insights on which mechanisms may be related to better outcomes (Gaume *et al.*, 2014; McCambridge and Rollnick, 2014; Miller and Moyers, 2015).

If current findings have shown mixed effects for BIs among young adults in the ER, and promising effects for MI among those intoxicated, more advanced research is needed on how to optimize this secondary prevention opportunity. This includes information on key intervention components, ideal quality of intervention delivery, and which particular sub-groups are most likely to benefit. We believe this is an important direction for intervention research with the potential to improve existing models (Longabaugh and Magill, 2011).

Recently, a review of studies investigating alcohol BI mechanisms (Gaume *et al.*, 2014) found these to be scarcely investigated even if some showed promise. Together with findings from the wider field of MI, they form possible hypotheses to investigate for the development of interventions for intoxicated young adults recruited in an ER setting. In particular, 6 components are listed below; these will compose the first intervention model further discussed and developed in Phase 1 of this project (see below).

1. Relational factors: empathy, acceptance, collaboration, and avoidance of confrontation

Relational factors can be significant determinants of addiction treatment outcome (Miller and Moyers, 2015). Empathy has been relatively well established as an active ingredient in the general psychotherapy literature (e.g. Norcross, 2011) and in addiction treatment (Moyers and Miller, 2013). Several interpersonal skills (e.g. acceptance, empathy, collaboration and support of client autonomy) have been related to client involvement in MI (Moyers *et al.*, 2005; Boardman *et al.*, 2006; Catley *et al.*, 2006) and to alcohol outcomes in BMI (Gaume *et al.*, 2008; Gaume *et al.*, 2009; Gaume *et al.*, *under review*). Reflective listening is an important technique to deepen understanding of patient's perspective (Miller and Rollnick, 2013). It has been related to more discussion of change (change talk) and enhanced outcomes (Barnett *et al.*, 2014; Magill *et al.*, 2014; Gaume *et al.*, *under review*). On the other hand, confrontation have been found to be particularly harmful, by decreasing client change talk, increasing resistance and sustain talk, and in some studies directly affecting client outcomes (Miller *et al.*, 1993; Apodaca and Longabaugh, 2009; Magill *et al.*, 2014; Gaume *et al.*, *under review*).

2. Personalized feedback

Early BI models have focused explicitly on feedback on risk or harm as a tool for instigating change (Bien *et al.*, 1993). Meta-analytic findings are supportive of the use of feedback (Bien *et al.*, 1993; Carey *et al.*, 2007; Carey *et al.*, 2012), with interventions including feedback having significantly better outcomes than intervention not including it. Studies that have experimentally investigated this question produced more mixed, but promising findings (Murphy *et al.*, 2004; Juarez *et al.*, 2006; Walters *et al.*, 2009; Cowell *et al.*, 2012). In a fundamental study of MI, providing feedback in a non-confrontational MI style doubled client change talk and halved resistance (Miller *et al.*, 1993).

3. Enhance discrepancy

To develop and resolve discrepancy between the individual's current behavior and broader life goals

and values is a core feature of MI (Miller and Rollnick, 2013). In one empirical study (McNally *et al.*, 2005), discrepancy measures were significantly increased following BMI and were correlated with alcohol outcomes among heavy-drinking college students. In an ER-based alcohol BI study, Walton *et al.* (2008) showed that injury attribution to alcohol moderated intervention effect, suggesting that highlighting the connection between alcohol and injury can augment intervention effectiveness. By extension, evocation of the current situation (alcohol intoxication, potentially alcohol related injury) in contrast with broader life goals and values might be an important mechanism of change.

4. Evoke change talk / Strengthen ability and commitment to change

MI has been described as a collaborative conversation style for strengthening a person's own motivation and commitment to change (Amrhein *et al.*, 2003) and central to it is the hypothesis that people are more likely to be persuaded by what they hear themselves say (Bem, 1972; Miller and Rollnick, 2013). Empirical support for change talk evocation as an active ingredient in MI has been accumulating (Miller and Rose, 2009; Moyers *et al.*, 2009; Magill *et al.*, 2014). Among the different dimensions of change talk, ability to change have been linked to confidence to change and self-efficacy, a central principle in MI (Miller and Rollnick, 2013); this dimension has been shown to predict enhanced outcomes in ER patients (Gaume *et al.*, 2008) and young adults (Baer *et al.*, 2008; Gaume *et al.*, 2013).

5. Change plan completion

Completion of a plan to change alcohol use is an MI component resulting in verbal statements of intention, and a written contract for behavior change (Magill *et al.*, 2010). Magill *et al.* (2010) showed that change plan completion was related to higher therapist MI skills and client change talk within the session. Lee *et al.* (2010) showed that good-quality change plan were associated with better outcomes, regardless of pre-intervention readiness to change.

6. More time: Longer sessions and/or booster sessions

Systematic reviews on alcohol BI and BMI have not clearly determined the optimal intervention length (O'Donnell *et al.*, 2014), even if more intensive interventions tended to yield overall more favorable results in ER-based intervention (Nilsen *et al.*, 2008). One study recently investigated the efficacy of 3 strategies of gradual intensity to address heavy drinking among injured patients (Field *et al.*, 2014). Findings showed that BMI plus telephone booster showed significant reductions in alcohol use and heavy drinking episodes compared with brief advice or BMI alone. This evidence suggests that longer interventions including booster sessions are more effective in an ER setting.

3.5 Dose Rationale

Based on the latter evidence that longer sessions and booster sessions are more effective in an ER setting (see 3.4, point 6, just above), we propose a motivational intervention of 20 to 60 minutes while in the ER, and up to 3 additional booster sessions by phone. It should be noted that MI relies on a focus on patients' autonomy (Miller et Rollnick 2013). The length of the session and the number of booster will thus be negotiated with the patient during the intervention. Referral to specialist services will also be proposed and discussed as part of the intervention.

3.6 Explanation for choice of comparator (or placebo)

Brief advice will be delivered in approximately 5 to 10 minutes. It will led by research clinicians and will consist of a standardized computer-based brief structured feedback and advice to cut down drinking, with referral to specialist services for the more severe patients (based on the Alcohol Use Disorders Identification Test score included in the baseline assessment). This intervention will be an adaptation of the electronic intervention www.alcooquizz.ch, guided by the clinician.

This condition is intended to control for exposure to non-study intervention effects and other non-specific study intervention effects including contact with a trained clinician, assessment, advice, referral and expectancies via an ethically acceptable alternative intervention.

Patients admitted while intoxicated in Lausanne University Hospital ER are currently receiving a brief alcohol intervention, including advice to cut down drinking and referral to specialist services, provided by a clinician trained in alcohol and addiction science. This brief intervention will be standardized to allow replication. We propose to adapt the computer-based brief structured intervention www.alcooquizz.ch, since it was showed that this intervention had a significant

secondary prevention effect among Swiss young men (Bertholet *et al.*, 2015).

Medical consultation (e.g. assessment and treatment of alcohol withdrawal symptoms, assessment and treatment of alcohol related pathologies) are not part of the brief interventions (neither BMI nor BA) and will be provided to all patients independently of study participation according to usual practice.

3.7 Risks / Benefits

3.7.1 Anticipated risks.

The risks of serious adverse consequences as a result of study participation are relatively low. It is possible that some participants will find the interviewing tiresome. They might also have concerns about confidentiality of sensitive information addressed during the intervention. The sensitive nature of some of the questions (e.g., alcohol-related) may cause participants discomfort.

Acknowledging these risks, care and attention will be given to implement the interviews using non-judgmental language and delivery. Second, participants will be informed of the sensitive and personal nature of the interview during the consent process and will be informed that they may refuse to participate or skip any questions they do not wish to answer. Participants will also be encouraged to contact one of the investigators/collaborators in case they may have any questions or concerns regarding the study or encounter any stress resulting from study participation.

Further, to protect the confidentiality of data, all interview data (including hair samples) will be identified with a unique personal identification number (PIN), which will be randomly assigned for the study. Master lists of names and PINs will be stored in locked file cabinets and password-protected computers with restricted access. Only allowed research staff will access these data.

3.7.2 Anticipated benefits and benefits/risks ratio.

There may be no direct benefit to participants. That being said, potential benefits to participants include the possibility to reduce alcohol consumption and related problem; participants also may benefit from clinical assessment, monitoring and referrals as necessary. They might also take advantage of an interview with a trained psychologist to think about their behaviors and their life values and better know themselves.

The current study is designed to develop and test a new BMI tailored to young adults. Potential benefits to society include increased knowledge about and possible development of a new and clinically significant BMI tailored to young drinkers. As explained above, the risks of adverse effects (i.e., psychological discomfort) are low in the current study and steps will be taken to minimize them. We therefore believe that the cost-benefit ratio if this study is appropriate.

3.8 Justification of choice of study population

All consecutive patients between the age of 18 and 35, admitted in the ER (for any cause) and having alcohol intoxication (>0.5 gram/liter BAC or clinical indication of alcohol intoxication) will be contacted by a member of the research staff.

Exclusion criterion will be a) life threatening conditions; b) detainees or medico-legal admissions; c) not being fluent in French; d) currently receiving another alcohol or substance use treatment; e) psychiatric or medical contra-indications preventing patients understanding informed consent, fulfilling questionnaires, and participating in the intervention (evaluated using an adaptation of the University of California, San Diego Brief Assessment of Capacity to Consent; McCormack *et al.*, 2014).

We target young adults as alcohol use among this population causes major harms. Harmful alcohol use results in approximately 2.5 million deaths annually and about 9% of these deaths are among young people between the ages of 15 and 29 (WHO, 2011). These deaths are mainly the result of alcohol-related motor vehicle accidents, homicides, suicides, and drowning. Heavy drinking episodes (i.e. drinking 6 standard drinks or more [>60 grams of pure alcohol] in a single occasion) and acute alcohol intoxication are associated with an increased risk of injuries, trauma, violence, risky sexual behaviors, and other negative health outcomes, especially among young adults (Rehm, 2011). Heavy drinking episodes during adolescence have also been related to an increased risk of

alcohol dependence, other substances abuse, psychiatric comorbidities, and social difficulties in adulthood (McCambridge *et al.*, 2011).

Additionally, the burden of unhealthy alcohol use on hospitals, and ERs in particular, is noteworthy (Pirmohamed *et al.*, 2000; Imlach *et al.*, 2011; Verelst *et al.*, 2012; Bertholet *et al.*, 2014). In Switzerland, ER admissions for alcohol intoxication have increased over the last decade, among all age groups (+11%), but particularly among adolescents and young adults (+57% among 10-23 years old; (Wicki and Stucki, 2014). At Lausanne University Hospital ER, the number of young adults between 18 and 30 years old admitted with a positive blood alcohol concentration (BAC ≥ 0.5 gram/liter) increased fourfold between 2000 and 2011 (from 125 to 544 cases). In 2011, 30% of all positive BACs recorded in this ER concerned young adults from 18-30 years old. Moreover, first results of a study on health care utilization by young adults admitted in this ER with alcohol intoxication showed that more than half of these patients were readmitted over the next 6 years, and 24% for a new alcohol intoxication episode.

While young adults accrue significant harm due to heavy alcohol use, studies of the natural history of alcohol use disorders have shown that the likelihood of such disorders is lower among younger individuals than it is among older individuals; if present, they are probably milder in severity (e.g. Schuckit *et al.*, 1995). Therefore, secondary prevention interventions are likely to be of substantial benefit with younger individuals (Sommers *et al.*, 2013). Reviews on strategies targeting alcohol use show that brief interventions (BI) are among the few effective preventive strategies and the most cost-effective strategy among individual-centered approaches (Babor *et al.*, 2010) and adolescents and young adults are particularly receptive to motivational methods because they include acceptance, avoidance of argumentation and hostile confrontation, and eschew giving lectures or ultimatums (Tevyaw and Monti, 2004).

4. STUDY OBJECTIVES

4.1 Overall Objective

The purpose of this project is to develop a new motivational intervention model for young adults admitted in the ER with alcohol intoxication and test the efficacy of this new model using a randomized controlled trial comparing it to a control condition receiving a minimal intervention (structured brief advice).

4.2 Primary Objective

The study seeks primarily to determine the effect of a motivational intervention compared to a control condition receiving a minimal intervention (structured brief advice) to decrease alcohol use and alcohol-related problems among young adults admitted in the ER with alcohol intoxication.

4.3 Secondary Objectives

Secondary objectives are to evaluate the mechanisms of the intervention effects by evaluating moderators of the effect (i.e. under which circumstances the effect is observed), mediators of the effect (i.e. how the effect is translated into actual behavior change), moderated mediation (i.e. if the effect is transmitted through the designated mediators only under specific circumstances), as well as by exploring intervention successes and failures using qualitative analyses.

4.4 Safety Objectives

Not applicable.

5. STUDY OUTCOMES

5.1 Primary Outcome

Primary outcomes measures will be

a) the number of Heavy drinking days over the last month (HDD, i.e. the number of days with 6 standard drinks or more, equivalent to 60 grams of pure alcohol or more) measured using a 30-day Timeline Follow-back technique (TLFB; Sobell and Sobell, 1995), see Chapter 9.2.1 for assessement details, and

b) the Short Inventory of Problems (SIP) total score (Kiluk *et al.*, 2013),

both measured over 3 follow-up times (3-, 6-, and 12-month after intervention).

These two measures were chosen as they are validated and largely used measures of change in alcohol use and alcohol-related problems, which are behaviour changes targeted by our motivational intervention.

5.2 Secondary Outcomes

Secondary outcomes measures will be

a) Weekly drinking amount (i.e. number of drinks per week, derived from the TLFB).

b) Frequency of alcohol-related consequences (derived from 9 alcohol-related consequences adapted from Wechsler *et al.*, 1994). Consequences will be measured over the same 3 follow-up times (3-, 6-, and 12-month after intervention). Consequences are another validated way of measuring alcohol-related problems.

c) SIP sub-dimension scores (Physical, Social, Intra-personal, Inter-personal, and Impulse control; Kiluk *et al.*, 2013). These scores will be measured over the same 3 follow-up times (3-, 6-, and 12-month after intervention). Sub-dimensions of the SIP instrument have been proposed as additional variables of interest. Intervention might have an effect on different sub-dimensions but not other.

d) Proportion of patients with hazardous or harmful drinking status (Alcohol use disorder identification test - AUDIT score>=8; Babor *et al.*, 2001) at 12-month follow-up. The AUDIT was developed by the WHO and is largely used to evaluate the effect of brief alcohol intervention. The reference period is 12 months and will thus be assessed only at this follow-up.

e) Proportion of patients who started alcohol treatment and of patients readmitted to the ER over the 12-month follow-up period. These will be measured at 3 follow-up times (3-, 6-, and 12-month after intervention). Effective referral to alcohol treatment and/or avoidance of alcohol-related ER readmission is a secondary objective of the tested intervention.

f) Proportion of patients who started alcohol treatment and of patients readmitted to the ER according to Lausanne University Hospital medical records. Participants will be asked for an additional, non-mandatory consent to have access to their administrative records at Lausanne University Hospital. This measure will be used as a confirmatory measure of patient self-report. It will also allow for evaluating participants lost to follow-up.

g) Heavy drinking according to Ethyl glucuronide (EtG) concentration in head hair. EtG has been presented as the only biomarker that can conclusively discriminate active heavy drinkers from social and non-drinkers (Crunelle *et al.*, 2014). In a study conducted at Lausanne University Hospital (Kharbouche *et al.*, 2012), cutoff values were determined to discriminate at-risk drinkers and heavy drinkers. Based on this study and others, cutoff values were proposed by the Society of Hair Testing (at-risk drinkers: >7 pg/mg EtG in hair; heavy drinkers : >30 pg/mg; Kintz, 2015). Participants in the present study will be asked for consent to take a 2-3 centimeters long strand of hair (diameter of pencil lead) for EtG testing at baseline, 6-, and 12-month follow-up. EtG testing will be a secondary, confirmatory outcome measure. Participants with head hair shorter than 2 centimeters or refusing hair sampling will not be excluded from the study. Research on cosmetic treatments of hair showed that permanent coloration, decoloration, and permanent wave might reduce EtG concentration (Kerekes and Yegles, 2013; Crunelle *et al.*, 2015). The occurrence of such treatments will be measured in the questionnaire and taken into account when interpreting EtG measures. Potential inconsistencies between this measure and other alcohol use measures will not lead in correction of alcohol use measures. Nonetheless, secondary analysis of the data to compare these different measures is planned and will be the focus of an article.

5.3 Other Outcomes of Interest

Additional measures for intervention mechanisms study will also be assessed at different time points (see table below) in order to conduct moderation and mediation analysis (see chapter 11.4.3 Secondary analysis).

	Baseline	Post-session	1-month	3-month	6-month	12-month
Alcohol use disorder severity (Babor *et al.*, 2001)	x					
Readiness Rulers (Rollnick, 1998)	x	x				
Actual-ideal discrepancy (McNally *et al.*, 2005)	x	x	x	x	x	
Alcohol-related expectancies (Ham *et al.*, 2005)	x		x	x	x	
Depression/Anxiety (Kroenke *et al.*, 2009)	x					
Attribution of injury to alcohol (Longabaugh *et al.*, 1995)	x					
Severity of ER admission and diagnosis (medical records)	x					
Quality of life (de Boer *et al.*, 2004)	x		x	x	x	
Patient rating of the treatment (Lee *et al.*, 2007)		x				
Working alliance inventory (Horvath and Greenberg, 1989)		x				
Trait reactance (Shen and Dillard, 2005)			x			

5.4 Safety Outcomes

No specific safety outcomes will be measured. See Chapter 10. Safety.

6. STUDY DESIGN

6.1 General study design and justification of design

6.1.1 Development and pre-test of a new intervention model (Aim 1)

This part corresponds to Aim 1 of the present project which consists of an iterative process aiming to develop and pre-test the intervention model through qualitative evaluation and refinement of the intervention tool. This process is planned over a 3-month period. The resulting intervention will be tested in the proposed randomized controlled trial (Aim 2).

As more thoroughly described above (see Chapter 3, Background), an initial intervention model was developed based on the existing literature on alcohol-related brief motivational interviewing (BMI) tailored to young adults as well as on the investigators' clinical and research experience. This first phase aims to pre-test the initial intervention model through iterative testing and refining by conducting one first round of experimental sessions with qualitative evaluations of the interventionists' and patients' experience (N=6), one round of international experts' consultation, and a second round of 6 experimental sessions with qualitative evaluations of the interventionists' and patients' experience.

6.1.2 Randomized controlled trial (Aim 2)

6.1.2.1 Treatments / intervention

The experimental treatment will be the brief motivational intervention (BMI) developed and pre-tested in the first phase of the project. BMI will be delivered while in the ER by a qualified research clinician (psychologist, clinical social worker, or nurse) and will last between 20 to 60 minutes. After the session, the interventionist sends a letter summing up the discussion (i.e., context, discussion, aims and encouragements) to the participant. Based on participant's agreement, booster session by phone will be conducted after **1 week** ~~**2 weeks**~~, 1 month, and 3 months.

"2 weeks" changed to "1 week", 28.11.2016 (before inclusion of first participant)

6.1.2.2 Population to be studied and the number of participants to be included

All consecutive patients between the age of 18 and 35, admitted in the ER (for any cause) and having alcohol intoxication (>0.5 gram/liter BAC or clinical indication of alcohol intoxication) will be contacted by a member of the research staff (see figure below). Exclusion criterion will be a) life threatening conditions; b) detainees or medico-legal admissions; c) not being fluent in French; d) currently receiving another alcohol or substance use treatment; e) psychiatric or medical contra-indications preventing patients understanding informed consent, fulfilling questionnaires, and participating in the intervention (evaluated using an adaptation of the University of California, San Diego Brief Assessment of Capacity to Consent; McCormack et al. 2014). Patients meeting inclusion criterion but being too intoxicated will be contacted by research staff once they have sobered up.

Targeted sample size is of 344 participants. This number was derived using a program for power analysis in longitudinal design (Rochon, 1998) which showed that a sample of N=172 patients per group (with attrition of 5-20% over follow-ups) will be required to detect small/medium effect sizes (.25), with power at .8, alpha at .05, and moderate autocorrelation dampening in GEE models for the primary outcomes. Primary analyses will use all data available from all participants included in the trial. Analysis will be performed in an intention to treat paradigm (i.e. participants having left the ER without finishing the intervention, and/or not receiving intervention booster sessions will be analyzed in the intervention group). In the event that we identify systematic mechanisms of missingness, we will explore varying techniques to impute missing data (Little and Rubin, 2002). (See also chapters 7.4 Criteria for withdrawal/discontinuation of participants, 11.4 Planned analyses, and 11.5 Handling of missing data and drop-outs).

6.1.2.3 Blinding

Participants will be blinded to which kind of interventions they are receiving. Clinicians cannot be blinded to the type of intervention they will have to deliver. All data collection through the eCRF will be undifferentiated according to the allocated study group, thus non-indicative to investigators and

follow-up interviewers.

6.1.2.4 *Kind of comparator(s), (e.g. placebo, no treatment, active drug, dose-response, historical and study configuration (parallel, cross-over)*

BMI will be tested against a control comparison minimal intervention (Brief advice).

Brief advice will be delivered in approximately 5 to 10 minutes. It will led by research clinicians and will consist of a standardized computer-based brief structured feedback and advice to cut down drinking, with referral to specialist services for the more severe patients (based on the Alcohol Use Disorders Identification Test score included in the baseline assessment).

6.1.2.5 *Method of assignment to treatment/intervention*

Simple randomisation (parallel, unstratified, 1:1 repartition ratio).

6.1.2.6 *Sequence and duration of all study periods*

See Randomized controlled trial flowchart below. Study inclusion is planned over 18 months.

See also chapter 9.1 Study flow chart / Table of study procedures and assessments.

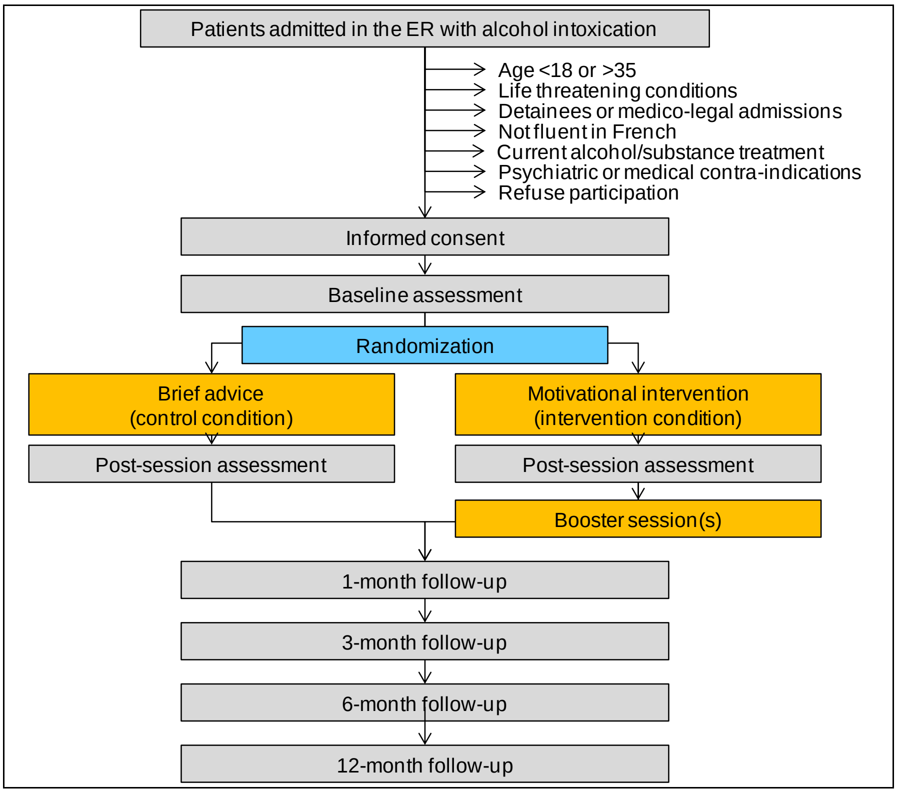

6.1.3 Mechanisms analysis (Aim 3)

Participants will be asked for additional consent to audio-record intervention sessions. Then, audio-recorded sessions will be analysed using psycholinguistic coding.

In addition, a random sub-sample of 20 BA and 20 BMI sessions will be transcribed and analyzed using thematic analysis and recursive abstraction techniques (Maxwell, 2005).

Also, semi-structured interviews will be conducted after the 1-month and 12-month follow-up questionnaire with another randomly selected 10 patients having received BA and 10 MI sessions. Topics addressed will be qualitative assessment of potential short-/long-term changes experienced during the follow-up period and their relation or not with the intervention received.

6.2 Methods of minimising bias

6.2.1 Randomisation

Randomization will be performed using an algorithm implemented in the same software used to collect baseline assessment (secuTrial). The unit will be the participants and there will be no stratification. There will be the exact same chance for each participant to receive intervention a) or b).

The algorithm will be developed and implemented by the Lausanne Clinical Trial Unit and will thus be totally independent of the research field staff and research investigators.

6.2.2 Blinding procedures

Participants will be blinded to which kind of interventions they are receiving. Both interventions will be presented as “clinical interviews”. They might realize that they had received a more or less long or intense intervention.

Clinicians cannot be blinded to the type of intervention they will have to deliver.

ER staff and related care providers will be blinded to intervention allocation. They will only know that the participant has received a secondary prevention intervention relative to alcohol use and consequences. They will provide care as usual to all participants.

Follow-up interviews will be conducted by research assistants, specifically trained for the research project, not implicated in baseline procedures, and blinded to patients’ group allocation and prior data. Weekly supervision will be organized with the PI to ensure data collection quality.

Data analysts will not be blinded.

6.2.3 Other methods of minimising bias

All questionnaires and randomization will be conducted and recorded on a secured electronic case report form (eCRF) managed by the Clinical Trial Unit, Lausanne, warranting data integrity, security, quality and traceability.

Weekly supervision will be organized with the PI to ensure data collection quality. Additional clinical supervision will be ongoing with Drs. Bertholet and Hugli to answer any clinical challenges encountered with study patients.

6.3 Unblinding Procedures (Code break)

Not applicable

7. STUDY POPULATION

7.1 Eligibility criteria

Participants fulfilling all of the following inclusion criteria are eligible for the study:

a) between the age of 18 and 35
b) admitted in the Lausanne University Hospital ER (for any cause) and having alcohol intoxication (>0.5 gram/liter BAC or clinical indication of alcohol intoxication)
c) Informed consent as documented by signature (see Appendix Informed Consent Form)

The presence of any one of the following exclusion criteria will lead to exclusion of the participant:

a) life threatening conditions;
b) detainees or medico-legal admissions;
c) not being fluent in French;
d) currently receiving another alcohol or substance use treatment;
e) psychiatric or medical contra-indications preventing patients understanding informed consent, fulfilling questionnaires, and participating in the intervention (evaluated using an adaptation of the University of California, San Diego Brief Assessment of Capacity to Consent; McCormack et al. 2014).

7.2 Recruitment and screening

The study will be conducted at the Lausanne University Hospital ER, Switzerland. According to administrative data over 7 weeks randomly selected between November 2012 and March 2013, intoxicated young adults are admitted almost only in the later part of the week. For economic reasons, we will limit study recruitment in the ER to Thursday to Sunday mornings from 7am to 2pm, covering 85% of eligible patients. Patients meeting inclusion criterion and having been treated outside this time frame will be contacted by phone and proposed to come to Lausanne University Hospital Alcohol Treatment Center to participate in the study.

All consecutive patients between the age of 18 and 35, admitted in the ER (for any cause) and having alcohol intoxication (>0.5 gram/liter BAC or clinical indication of alcohol intoxication) will be contacted by a member of the research staff during study period. Patients meeting inclusion criterion but being too intoxicated will be contacted by research staff once they have sobered up.

First, research staff will conduct a screening process to evaluate inclusion and exclusion criterion (see Chapter 7.1 above). In case only exclusion criteria e) is not fulfilled (i.e. psychiatric or medical contra-indications preventing patients understanding informed consent, fulfilling questionnaires, and participating in the intervention; evaluated using an adaptation of the University of California, San Diego Brief Assessment of Capacity to Consent – UBACC; McCormack et al. 2014), research staff will come back later to re-assess UBACC and complete further study process if the UBACC score is positive.

For those included, baseline assessment questionnaire will then be conducted in the ER, in a separate space providing confidentiality and using a tablet-based electronic questionnaire administered by a research clinician. The questionnaire will be short (about 5 minutes), first as patients might be hung-over as a result of alcohol intoxication, and second to reduce reactivity to alcohol assessment which has been shown as a source of bias (Gallen, 1974; McCambridge & Kypri, 2011).

7.3 Assignment to study groups

After completing the computer-based assessment, the software will automatically randomize participants into 2 equivalent groups: a) Brief motivational intervention (experimental condition) and b) Brief advice (control condition).

7.4 Criteria for withdrawal / discontinuation of participants

Each participant will be informed that study participation is voluntary and he/she may withdraw from

the study at any time.

Participants will be considered included in the study when signing informed consent.

Participants withdrawing before starting interventions will be replaced to obtain sufficient power (N=344 participants having received intervention). Participants withdrawing after having started brief intervention (either experimental or control) will not be replaced.

Investigators will not withdraw included participants from the study.

8. STUDY INTERVENTION

8.1 Identity of Investigational Products (treatment / medical device)

8.1.1 Experimental Intervention (treatment / medical device)

The experimental treatment will be the brief motivational intervention (BMI) developed and pre-tested in the first phase of the project.

8.1.2 Control Intervention (standard/routine/comparator treatment / medical device)

The control intervention will be a minimal intervention - *Brief advice*.

8.1.3 Packaging, Labelling and Supply (re-supply)

Not applicable.

8.1.4 Storage Conditions

Not applicable.

8.2 Administration of experimental and control interventions

8.2.1 Experimental Intervention

BMI will be delivered while in the ER by a qualified research clinician (psychologist, clinical social worker, or nurse) and will last between 20 to 60 minutes.

The intervention will use 3 main ingredients, in particular a) relational factors (e.g., empathy, acceptance, collaboration, avoidance of confrontation), b) evoking participants' change talk and strengthening their ability and commitment to change, and c) build a relationship significant in the long-run (e.g., including follow-up booster sessions). Overall, the intervention aims at increasing participants' motivation to change their drinking behaviours by enhancing discrepancy between their current behaviour and their broader life goals and values. The intervention focuses on helping participants to resolve the latter discrepancy through evoking and planning behaviour change. When necessary, interventionist will discuss and facilitate referral to alcohol treatment.

After the session, the interventionist sends a letter summing up the discussion (i.e., context, discussion, aims and encouragements) to the participant. Based on participant's agreement, booster session by phone will be conducted after 2 weeks, 1 month, and 3 months.

8.2.2 Control Intervention

The control intervention will be a minimal intervention - *Brief advice (BA)* - delivered in approximately 5 to 10 minutes while in the ER by the same qualified research clinicians (psychologist, clinical social worker, or nurse). It will consist of a standardized computer-based brief structured feedback and advice to cut down drinking, with referral to specialist services for the more severe patients (based on the Alcohol Use Disorders Identification Test score included in the baseline assessment).

8.3 Dose / Device modifications

Not applicable.

8.4 Compliance with study intervention

Interventionists will be clinicians experienced in counseling and liaison for alcohol use disorder (psychologists, clinical social workers, or nurses). They will not be ER staff; firstly to avoid additional workload for the ER staff; and secondly as recent research showed that liaison consults and

interventions delivered by addiction specialists were more appropriate and effective (Schwan et al. 2012; Dunn et al. 2014).

Clinicians will be selected to have at least 1 year experience with alcohol use disorder and MI, as experienced therapists and MI skills have been shown as important predictors of better alcohol BMI outcomes (Gaume et al. 2009; Gaume et al. 2014a, Gaume et al. 2016). They will be specifically trained to provide the present intervention. Proficiency in MI will be assessed during training MI sessions with standardized patients, coded using the MI Treatment Integrity coding scheme (MITI; Moyers et al. 2005a).

Ongoing supervision will be provided throughout the project with interventions reviewed by a senior clinician expert in MI and feedback given to the clinician during biweekly supervision meetings.

BMI integrity will be assessed by the supervisor using the MITI. Adherence to the BA model and to the new BMI model will be assessed using an ad-hoc Components Inventory developed for this project after Phase 1. This instrument will be developed similar to the Process Rating Scale (Tober et al. 2008) used in the United Kingdom Alcohol Treatment Trial (UKATT, i.e. a check list of intervention components with 2 items by component: a) the extent to which the component was performed, and b) the quality of this performance). Interventionists performing interventions under acceptable thresholds will be notified during the next supervision, and will be replaced if practice is judged consistently below the identified levels over time.

8.5 Data Collection and Follow-up for withdrawn participants

Investigators will not withdraw participants from the study. Participants may withdraw from the study at any time and might be lost to follow-up.

Participants withdrawing from the trial will not be contacted for subsequent questionnaires. However, if they consented to within the initial consent process, their hospital administrative records will be consulted to have an objective, non self-report measure of readmission in the ER and alcohol treatment at 12 months post baseline.

8.6 Trial specific preventive measures

Not applicable.

8.7 Concomitant Interventions (treatments)

Not applicable.

8.8 Study Drug / Medical Device Accountability

Not applicable.

8.9 Return or Destruction of Study Drug / Medical Device

Not applicable.

9. STUDY ASSESSMENTS

9.1 Study flow chart(s) / table of study procedures and assessments

Phase 1 – Development (N=12)	
Visit	1 (in the ER)
Patient Information and Informed Consent	x
Brief motivational intervention with research clinician	x
Qualitative interview with research collaborator	x

Phase 2 – Randomized controlled trial (N=342)									
Study Periods	Baseline procedure	Intervention	Post-intervention	Follow-up					
Time (months)	0			1	3	6		12	
Visit	1			2	3	4	5	6	7
Location (ER=Emergency Room, T=telephone, A=Alcohol Treatment Center)	ER			T	T	T	A	T	A
Expected duration (minutes)	15	BMI: 20-60 BA: 5-10	5	15-20	20-30	20-30	5	15-20	5
In- /Exclusion Criteria	x								
Patient Information and Informed Consent	x								
Demographics (questionnaire)	x								
Primary outcomes variables (questionnaires)	x			x	x	x		x	
Secondary outcomes variables (questionnaires)	x			x	x	x		x	
Other outcomes of interest (questionnaires)	x		x	x	x	x			
Randomisation	x	x							
Intervention with research clinician:		x							
Head hair sample (only if hair>2 cm and additional consent)			x				x		x
Consultation of administrative and medical records (only if additional consent)								Not in presence of patient	

Phase 3 – Mechanisms analysis	
	Timing
15-20 minute qualitative semi-structured interview after follow-up questionnaire (N=10 participants in BMI and 10 in BA groups, randomly selected and consenting to continue the follow-up questionnaire with a discussion on their experience).	At the end of 1-month follow-up

15-20 minute qualitative semi-structured interview after follow-up questionnaire (N=10 participants in BMI and 10 in BA groups, randomly selected and consenting to continue the follow-up questionnaire with a discussion on their experience).	At the end of 12-month follow-up
Psycholinguistic coding of audio-recorded interventions (N=all interventions with patient's consent for audio-recording and no technical problems)	End of inclusion, not in presence of patient
Qualitative analysis of transcribed intervention sessions (N=10 BMI and 10 BA, randomly selected among all interventions with patient's consent for audio-recording and no technical problems)	End of inclusion, not in presence of patient

9.2 Assessments of outcomes

9.2.1 Assessment of primary outcome

Primary outcomes measures will be

a) the number of Heavy drinking days (HDD, i.e. the number of days with 6 standard drinks or more, equivalent to 60 grams of pure alcohol or more) over the last month, and

b) the Short Inventory of Problems (SIP) total score (Kiluk *et al.*, 2013),

both measured over 3 follow-up times (3-, 6-, and 12-month after intervention).

HDD will be measured using a 30-day Timeline Follow-back technique (TLFB; Sobell and Sobell, 1995). This technique will be used by a trained interviewer and consists in asking participants to retrospectively estimate their daily alcohol consumption over a time period (i.e., 30 days in the current study) prior to the interview. To help participants provide retrospective estimates of their daily alcohol use, the interviewer uses a calendar with temporal anchors such as holidays, week-end days, local festivals or parties, participant's activities, etc.

The SIP is a validated 15-item scale to evaluate alcohol-related problems.

9.2.2 Assessment of secondary outcomes

Secondary outcomes measures will be

a) Weekly drinking amount (i.e. number of drinks per week, derived from the TLFB).

b) Frequency of alcohol-related consequences (derived from 4 alcohol-related consequences adapted from Wechsler *et al.*, 1994). Consequences will be measured over the same 3 follow-up times (3-, 6-, and 12-month after intervention).

c) SIP sub-dimension scores (Physical, Social, Intra-personal, Inter-personal, and Impulse control; Kiluk *et al.*, 2013). These scores will be measured over the same 3 follow-up times (3-, 6-, and 12-month after intervention).

d) Proportion of patients with hazardous or harmful drinking status (Alcohol use disorder identification test - AUDIT score>=8; Babor *et al.*, 2001) at 12-month follow-up. The reference period is 12 months and will thus be assessed only at this follow-up.

e) Proportion of patients who started alcohol treatment and of patients readmitted to the ER over the 12-month follow-up period. These will be measured by asking the participant at 3 follow-up times (3-, 6-, and 12-month after intervention).

f) Proportion of patients who started alcohol treatment and of patients readmitted to the ER according to Lausanne University Hospital medical records. Participants' records over the 12 months following inclusion visit will be consulted by a research assistant at the end of the data collection process.

g) Heavy drinking according to Ethyl glucuronide (EtG) concentration in head hair. EtG has been presented as the only biomarker that can conclusively discriminate active heavy drinkers from social and non-drinkers (Crunelle *et al.*, 2014). Participants in the present study will be asked for consent to take a 2-3 centimeters long strand of hair (diameter of pencil lead) for EtG testing at baseline, 6-, and 12-month follow-up. Laboratory testing of EtG will be conducted at the end of the data collection process.

9.2.3 Assessment of other outcomes of interest

Other variables of interest will be variables for mechanisms of change analysis (see Chapter 5.3 for a list). These variables will be assessed by trained research clinicians/assistants using validated scales.

9.2.4 Assessment of safety outcomes

Not applicable.

9.2.5 Assessments in participants who prematurely stop the study

Participants withdrawing from the trial will not be contacted for subsequent questionnaires. However, if they consented to within the initial consent process, their hospital administrative records will be consulted (readmission in the ER and alcohol treatment) at 12 months post baseline.

9.3 Procedures at each visit

9.3.1 Day 1 – Inclusion, Baseline procedures, Intervention (in the ER)

A. Inclusion/Exclusion criteria from medical file/ER exam

- Age
- Blood alcohol concentration at admission (test made by ER staff)
- Life threatening conditions
- Detainees or medico-legal admissions
- Medical or psychiatric contraindications preventing contact with patient

B. Inclusion/Exclusion criteria assessed by research staff

- Being fluent in French
- Not currently receiving another alcohol or substance use treatment

C. Consent process

- Information sheet
- No psychiatric or medical contra-indications preventing patients understanding informed consent, fulfilling questionnaires, and participating in the intervention (evaluated using an adaptation of the University of California, San Diego Brief Assessment of Capacity to Consent; McCormack et al. 2014)
- Signing consent form

D. Contact information

- Name, Address, Phone

E. Baseline questionnaire

- Socio-demographic questions
- Alcohol Use Disorder Identification Test
- Substance use
- Attribution of ER admission to alcohol
- Readiness Rulers
- Alcohol-related expectancies
- Actual-ideal drinking discrepancy
- Depression
- Quality of Life

F. Randomization

- Software-based automatic allocation to study group (BMI vs BA)

G. Intervention

H. Post-session questionnaire

- Patient rating of the treatment (paper/pencil questionnaire to be placed in a sealed envelope by the participant and later entered by research assistant to avoid that research

clinicians have access to participant's rating of the session)

- Readiness Rulers
- Actual-ideal drinking discrepancy

I. Counselor's rating of the treatment

- Working alliance inventory

J. Head hair sampling for Ethyl Glucuronide concentration analysis

- Additional information and consent procedure
- Sampling of 2-3 centimeters long strand of hair (diameter of pencil lead)

K. Additional information on admission (medical records, not in presence of patient)

- Admission and discharge time
- Diagnosis
- Use of restraints
- Passage in resuscitation area
- Orientation at ER discharge

9.3.2 Month 1, by phone

- Alcohol use (30-day timeline follow-back)
- Readiness to change questionnaire
- Alcohol Abstinence Self-efficacy Scale – Reduced drinking
- Alcohol-related expectancies
- Actual-ideal drinking discrepancy
- Substance use
- Quality of life
- Alcohol treatment
- Trait reactance (Hong) – 14 items
- Alcohol treatment

9.3.3 Month 3, by phone

- Alcohol use (30-day timeline follow-back)
- Alcohol problems and consequences
- Readiness to change questionnaire
- Alcohol Abstinence Self-efficacy Scale – Reduced drinking
- Alcohol-related expectancies
- Actual-ideal drinking discrepancy
- Substance use
- Quality of life
- ER readmission
- Alcohol treatment

9.3.4 Month 6, by phone

- Alcohol use (30-day timeline follow-back)
- Alcohol problems and consequences
- Readiness to change questionnaire
- Alcohol Abstinence Self-efficacy Scale – Reduced drinking
- Alcohol-related expectancies
- Actual-ideal drinking discrepancy
- Substance use
- Quality of life
- ER readmission
- Alcohol treatment
- Set appointment for head hair sampling (if baseline consent)

9.3.5 Month 6, hair sampling (at Alcohol Treatment Centre)

- Sampling of 2-3 centimetres long strand of hair (diameter of pencil lead)

9.3.6 Month 12, by phone

- Alcohol use (30-day timeline follow-back)
- Alcohol problems and consequences
- Alcohol Use Disorder Identification Test
- Substance use
- Quality of life
- ER readmission
- Alcohol treatment
- Set appointment for head hair sampling (if baseline consent)

9.3.7 Month 12, hair sampling (at Alcohol Treatment Centre)

- Sampling of 2-3 centimetres long strand of hair (diameter of pencil lead)

9.3.8 Month 12, not in presence of the participant

If the participant consented to have their administrative and medical records consulted by the study team, a research collaborator specifically trained will access CHUV's administrative and medical records to collect participants' information regarding

- ER readmission
- Alcohol treatment

over the 12-month follow-up period.

10. SAFETY

This study falls into the “Other Clinical Trial”, in the Category of risk A.

The current clinical trial is neither a trial of therapeutic products or transplant products, nor a trial of transplantation. The health-related intervention investigated (i.e., a brief alcohol-related motivational interviewing) entails only minimal and temporary risks and burdens on participants’ health (i.e., well-being). The health-related intervention investigated (i.e., a brief alcohol-related motivational interviewing) is recognized as standard in guidelines prepared in accordance with internationally accepted quality criteria. For example, a recent report by the European Monitoring Centre for Drugs and Drug Addiction (2016) concluded that the potential benefits of brief interventions for alcohol and drug users needed to be further studied, yet the feasibility of such interventions, the absence of reported adverse effects and the potential cost-effectiveness suggested that brief interventions could be considered as part of the training for emergency department healthcare professionals.

The risks of serious adverse consequences as a result of study participation are relatively low. It is possible that some participants will find the interviewing tiresome. They might also have concerns about confidentiality of sensitive information addressed during the intervention. The sensitive nature of some of the questions (e.g., alcohol-related) may cause participants discomfort. Acknowledging these risks, care and attention will be given to implement the interviews using nonjudgmental language and delivery. Second, participants will be informed of the sensitive and personal nature of the interview during the consent process and will be informed that they may refuse to participate or skip any questions they do not wish to answer. Participants will also be encouraged to contact one of the investigators/collaborators in case they may have any questions or concerns regarding the study or encounter any stress resulting from study participation. In case participants would find participating in the study too tiresome or intrusive, they will be free to easily withdraw from the study by asking the research clinician to stop the interview (baseline and booster interventions), or deliberately not answering or stopping the phone call (follow-up questionnaires).

11. STATISTICAL METHODS

11.1 Hypothesis

The primary aim of the randomized controlled trial is to test the efficacy of the brief motivational intervention model developed in the first part of this project by comparing it to a control condition receiving a minimal intervention (structured brief advice).

The Null hypothesis is that both interventions have comparable effects in reducing

a) the number of Heavy drinking days (HDD, i.e. the number of days with 6 standard drinks or more, equivalent to 60 grams of pure alcohol or more, over the last month), and

b) alcohol-related problems as measured by the Short Inventory of Problems (SIP) total score,

over 3 the follow-up times (3-, 6-, and 12-month after intervention).

The Alternative hypothesis is that participants in the Brief motivational intervention group (experimental condition – intervention model developed in the first phase of the project) reduce

a) the number of Heavy drinking days (HDD, i.e. the number of days with 6 standard drinks or more, equivalent to 60 grams of pure alcohol or more, over the last month), and

b) alcohol-related problems as measured by the Short Inventory of Problems (SIP) total score,

more then participants in the control group (structured brief advice) over the 3 follow-up times (3-, 6-, and 12-month after intervention).

11.2 Determination of Sample Size

Based on studies with similar population (intoxicated young adults), setting (ER), and intervention (MI or BMI), we anticipate small to medium effects in favour of participants in the BMI group on alcohol use measures and alcohol-related problems (Monti et al. 1999; Smith et al. 2003; Spirito et al. 2004; Sommers et al. 2006; Monti et al. 2007).

Using a program for power analysis in longitudinal design (Rochon, 1998) a sample of N=172 patients per group (with attrition of 5-20% over follow-ups) will be required to detect small/medium effect sizes (.25), with power at .8, alpha at .05, and moderate autocorrelation dampening in GEE models for the primary outcomes.

The total sample size will thus be of 344 participants (1:1 ratio).

11.3 Statistical criteria of termination of trial

Not applicable.

11.4 Planned Analyses

11.4.1 Datasets to be analysed, analysis populations

Primary analyses of the efficacy of the intervention (randomized controlled trial) will use all data available from all participants included in the trial. Analysis will be performed in an intention to treat paradigm (i.e. participants having left the ER without finishing the intervention, and/or not receiving intervention booster sessions will be analyzed in the intervention group).

Secondary analysis of mechanisms (Aim 3 of the project) will use data available for the corresponding analyses (e.g. participants with audio-recorded sessions for psycholinguistic coding; randomly selected sub-sample for qualitative analyses). Also, moderation analyses are designed as part of the mechanism analyses. For these, the relative efficacy of the BMI will be tested as an interaction with participants' characteristics, such as age, gender, alcohol problems severity, readiness to change, or personality traits.

11.4.2 Primary Analysis

We will test intervention effects over time by comparing groups on the 2 primary and 6 secondary outcomes. For repeated measures, analyses will be conducted using generalized estimating equations (GEE) with robust standard error estimate (Liang and Zeger, 1986). This approach offers an extension of regression analysis to the case of correlated observations over time; it allows for specification of the assumed correlation structure as well as the appropriate link functions to handle dependent variables with a normal distribution, dichotomous outcomes, count data (Poisson), and over-dispersed count data (negative binomial). Models will all be adjusted for the baseline AUDIT score. For outcomes measured at 12-month only, analyses will be conducted using baseline adjusted regression models.

Analyses will be performed at the completion of data collection.

Analyses will be performed by the study PI (J. Gaume), in collaboration with research collaborator V. Grazioli, and consulting from study co-Is (N. Bertholet, J.-B. Daeppen, O. Hugli) and international collaborators (J. McCambridge, M Magill).

11.4.3 Secondary Analyses

Analyses of intervention mechanisms (Aim 3 of the project) will included the following analyses:

Moderation. Separate GEE models will test the main effect of treatment on outcome, followed by the effect of each moderator variable, and then the interaction of the treatment with each moderator variable.

Mediation. To examine mediators of the main effect of the MI intervention on outcomes, we will use Model 4 of the PROCESS algorithm developed by Hayes (2013). Here, regression analyses are used to test path *a* (the effect of the treatment condition on the hypothesized mediators) and path *b* (the effect of each respective mediator on outcomes, while also controlling for the treatment effect on outcomes). Then indirect effects are estimated using bootstrapping, a nonparametric resampling procedure that does not impose the assumption of normality of the sampling distribution (Hayes, 2013; Preacher & Hayes, 2008).

Moderated mediation aims at measuring if the effect is transmitted through the designated mediators only under specific circumstances. *S*everal models of the PROCESS algorithm (e.g. 7, 9, 58) allow calculation of moderated mediation (also known as conditional indirect effects) (Hayes, 2013) (Hayes, 2013) (Hayes, 2013) (Hayes, 2013) (Hayes, 2013) (Hayes, 2013) (Hayes, 2013) (Hayes, 2013) (Hayes, 2013) (Hayes, 2013) (Hayes, 2013). These different models specify which path (*a*, *b*, both) of the mediated relationship are moderated. As in mediation, bootstrapping is used to derive conditional indirect effects.

Qualitative analyses. In addition to the quantitative analysis of interventions' mechanisms exposed above, we will use qualitative techniques to analyze intervention and follow-ups feedback. A random sub-sample of 20 BA and 20 MI sessions will be transcribed and analyzed using thematic analysis and recursive abstraction techniques (Maxwell, 2005). In addition, semi-structured interviews will be conducted after the 1-month and 12-month follow-up questionnaire with another randomly selected 10 patients having received BA and 10 MI sessions. Topics addressed will be qualitative assessment of potential short-/long-term changes experienced during the follow-up period and their relation or not with the intervention received.

Regarding hypotheses, the proposed research will be one of the first prospective study designed to evaluate the mechanisms of alcohol BMI outcomes, which is in line with recently recommended priorities in clinical trial design (Longabaugh and Magill, 2011). Specifically, we will analyze treatment effects within a larger conceptual framework of mechanisms of behavior change (see Figure below). The mechanisms investigated here are all indicated as promising candidate variables in the seminal monograph on factors impacting addiction treatment effects recently published by Miller and Moyers (2015). In this model, the main effect (red arrow) between treatment and change in alcohol use (distal outcome) is explained (or mediated) by therapists' and patients' within-session behaviors (e.g. MI skills and change language), by the patients' experience of the treatment, and by patient proximal cognitive or behavioral changes (e.g. increased self-efficacy, readiness to change, Longabaugh *et al.*, 2005; Moos, 2007). Patient behaviors during treatment might influence their experience of the treatment and thus their outcome, but also influence therapist behaviors (as indicated by bidirectional arrow; e.g. Moyers and Martin, 2006; Gaume *et al.*, 2010). Finally, what happens during the session (therapist and patient behaviors) may be influenced by several moderating factors (e.g. Karno and Longabaugh, 2007; Gaume *et al.*, 2009; Gaume *et al.*, 2016).

These factors mainly are the therapist and patient characteristics, such as age, gender, therapist clinical experience, patient alcohol problems severity, readiness to change, or personality. (Note: our design will not permit tests of therapist characteristics which will be underpowered).

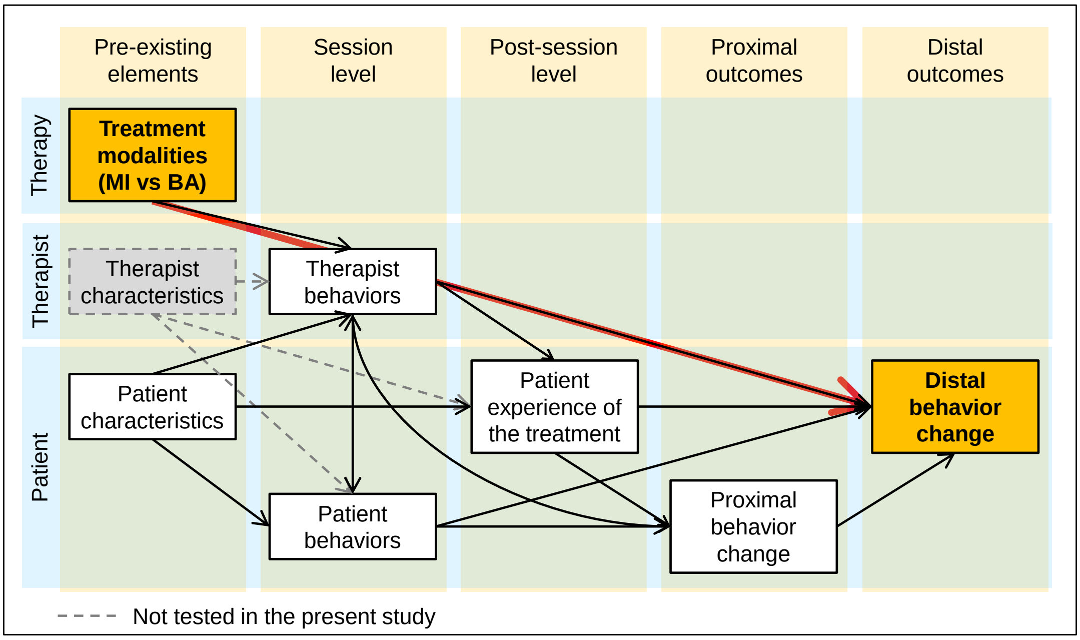

Quantitative analyses will be performed by the study PI (J. Gaume), in collaboration with research collaborator V. Grazioli, and consulting from study co-Is (N. Bertholet, J.-B. Daeppen, O. Hugli) and international collaborators (J. McCambridge, M Magill).

Qualitative Analyses will be performed by research collaborator V. Grazioli and S. Paroz, with consulting from study PI (J. Gaume), co-Is (N. Bertholet, J.-B. Daeppen, O. Hugli), and international collaborators (J. McCambridge, M Magill).

Analyses will be performed at the completion of respective data collection, but at least after the completion of all interventions (e.g. qualitative analysis of participants' experience at 1-month follow-up will not be performed before completion of all interventions in order not to influence intervention content).

11.4.4 Interim analyses

There will be no interim analyses.

11.4.5 Safety analysis

Not applicable (see Chapter 10. Safety).

11.4.6 Deviation(s) from the original statistical plan

Any deviation(s) from the original statistical plan will be described and justified in the final report.

11.5 Handling of missing data and drop-outs

Preliminary analyses will examine variable distributional properties, correlations, and patterns of missing data to inform data-analytic procedures. In the event that we identify systematic mechanisms of missingness, we will explore varying techniques to impute missing data (Little and Rubin, 2002); primary analyses will use all available data in an intent-to-treat paradigm.

12. QUALITY ASSURANCE AND CONTROL

12.1 Data handling and record keeping / archiving

12.1.1 Case Report Forms

All trial data (demographic informations, questionnaires, randomization, ...) of each participant will be directly recorded in an electronic case report form (eCRF, secuTrial software) independently managed by the Clinical Trial Unit, Lausanne, warranting data integrity, security, quality and traceability. Only authorized person (delegated by the principal investigator) will be allowed to proceed to eCRF entries.

The only exception is the patient rating of the treatment questionnaire which is a 12-item questionnaire assessing participant's rating of the intervention right after the intervention. This questionnaire will be self-reported using a paper/pencil questionnaire to be placed in a sealed envelope by the participant and later entered by research assistant to avoid that research clinicians have access to participant's rating of the session.

12.1.2 Specification of source documents

Signed informed consent forms, Contact information forms, and Patient rating of the treatment questionnaires are considered paper source documents and will be stored in the investigator site file in locked cabinets at the Alcohol treatment center, Lausanne university hospital.

Socio-demographic data and baseline/follow-up dates will also be considered source documents and will be directly collected in the e-CRF.

12.1.3 Record keeping / archiving

Paper data and audio-recording will be brought back to the Alcohol Treatment Center by the research clinicians at the end of the inclusion period and immediately secured in file locked cabinets (paper data) or electronically stored on secured servers (audio-recordings).

All paper study data filed in the investigator site file/ trial master file (ISF/TMF) will be archived for a minimum of 10 years after study termination or premature termination of the clinical trial in locked cabinets at the Alcohol treatment center, Lausanne university hospital.

Audio-recordings will be stored using only the participant's identification code. Audio-recordings will be electronically stored on secured servers protected by a password accessible only to psycholinguistic coding staff, clinical supervisers, and qualitative research analysts.

Electronic archives will be stored on secured servers for a minimum of 10 years also.

12.2 Data management

12.2.1 Data Management System

All questionnaires and randomization will be conducted and recorded directly on a secured electronic case report form (eCRF, secuTrial software) independently managed by the Clinical Trial Unit, Lausanne, warranting data integrity, security, quality and traceability.

12.2.2 Data security, access and back-up

Data will be entered directly in the eCRF by research clinicians (baseline procedures, intervention) and research assistants (follow-up questionnaires).

Access to raw data will be granted to the study PI or to research collaborators explicity allowed to access data by the study PI.

Access to contact data will be granted to the research collaborator in charge of compiling lists for follow-up contacts (Dr. Grazioli). These lists will be given to research assistants in charge of follow-up questionnaires.

Access to all other data will be granted to the study PI or to research collaborators explicity allowed

to access data by the study PI, e.g. to extract data for analysis.

No one will be permitted to alter data, except a) respective research clinicians/assistants having noticed an error in their own data entry, or b) the study PI or a research collaborator explicity allowed to by the study PI in case an error has been noted during monitoring or electronic validation. All data alteration will be traced in the software (secuTrial) and will require a justification documented in a comment field. When complete, each eCRFwill be validated by the study PI using dedicated entry fields (i.e. a check box “I hereby confirm that data entered are correct and complete”, and entry fields for name and date). The built-in traceability of the eCRF software (secuTrial) will guarantee that this signature is valid (PI login/password, date and time of entry).

Backup of electronic data are built-in in the eCRF software (secuTrial) and on CHUV servers.

12.2.3 Analysis and archiving

Data will be extracted for analysis by the study PI or a research collaborator explicity allowed to access data by the study PI. Data will be extracted to one or several databases compiled on statistical packages (e.g. Stata, SPSS, SAS, R).

Databases will be stored on electronic folders secured on the CHUV servers and protected by passwords. Access to the datasets will be granted to research collaborator explicity allowed to access data by the study PI.

Databases used for primary analyses as well as secondary analyses later published will be stored on electronic folders secured on the CHUV servers and protected by passwords for 10 years. Databases used for temporary analyses or secondary analyses not retained will be destroyed.

12.2.4 Electronic and central data validation

Data validity, coherence, and completeness will be assessed at several steps. First, control rules will be implemented in the data entry software (secuTrial). Second, coherence of study plan and data collection will be regularly assessed by the study PI thoughout the study and discussed during weekly supervision of research staff. Then, monitoring performed by the Clinical Trial Unit, Lausanne (see below) will encompass monitoring of data coherence and completeness, as well as systematic documentation of alteration in the data. Finally, coherence and completeness will be checked when the data will be exported to statistical packages for analyses.

12.3 Monitoring

Monitoring will be performed according to ICH Good Clinical Practice (GCP) by the Clinical Trial Unit, Lausanne. Following a Monitoring Plan and written SOPs, the monitors will verify that the clinical trial is conducted and data are generated, documented and reported in compliance with the protocol, GCP and the applicable regulatory requirements. The principal investigator will provide direct access to all trial related source data/documents and reports for the purpose of monitoring and will answer monitors’ questions during monitoring visits.

12.4 Audits and Inspections

No trial audit is planned. However in case of audit and/or inspection by the responsible ethics committee, the study documentation and source data/documents will be accessible to auditors/inspectors and questions will be answered during audits/inspections. All involved parties will keep the participant data stricty confidential.

12.5 Confidentiality, Data Protection

Data protection and confidentiality will be guaranteed. Direct access to source documents will be permitted for purposes of monitoring (12.3), audits and inspections (12.4).

No printed nor online reports will be published with participants’ name or possible identification. Audio-recordings will in principe not be used in public, but in case it would, only excerpts not divulging participants identity would be used and voice would be distorted.

Deidentified datasets, protocol, and statistical code will be available for co-investigators, international collaborators, and close collaborators or students to run potential additional secondary analyses. This material might also be made available for consultation during peer-review processes for study publication.

12.6 Storage of biological material and related health data

Coded head hair samples will be stored in locked cabinets at the Alcohol Treatment Center, Lausanne University Hospital, until completion of data collection. Samples will then be transferred to the Forensic Toxicology and Chemistry Unit at the Lausanne and Geneva Universities Centre of Legal Medicine where analyses will be processed.

Samples will be destroyed at the end of the study.

13. PUBLICATION AND DISSEMINATION POLICY

Our findings will be presented during scientific conferences at all phases (intervention development, efficacy study, mechanisms analyses, and model finalization). Several articles will be published in leading peer-review journals in the respective fields: 1 or 2 on intervention development, 1 reporting the results of the RCT, 1 technical paper on EtG concentration in head hair, 2 or 3 on mechanisms analyses, 1 on qualitative analyses of intervention and follow-up semi-structured interviews, and 1 on model finalization.

Anonymized data will be available for co-investigators, international collaborators, and close collaborators or students to run potential additional secondary analysis.

A press conference and press release will be carried out at the end of the project to present the research process and findings to the lay public.

The study PI will coordinate analyses, scientific presentations in conference, and journal articles. Authorship will follow standard guidelines (e.g. International Committee of Medical Journal Editors recommendations).

14. FUNDING AND SUPPORT

14.1 Funding

Swiss National Science Foundation, Grant 105319_163123.

14.2 Other Support

None.

15. INSURANCE

Not applicable (Category A).

16. REFERENCES

1. Declaration of Helsinki, Version October 2013, (http://www.wma.net/en/30publications/10policies/b3/index.html)
2. International Conference on Harmonization (ICH, 1996) E6 Guideline for Good Clinical Practice. (http://www.ich.org/fileadmin/Public_Web_Site/ICH_Products/Guidelines/Efficacy/E6_R1/Step4/E6_R1__Guideline.pdf)
3. International Conference on Harmonization (ICH, 1997) E8 Guideline: General Considerations for Clinical Trials http://www.ich.org/fileadmin/Public_Web_Site/ICH_Products/Guidelines/Efficacy/E8/Step4/E8_Guideline.pdf)
4. Humanforschungsgesetz, HFG Bundesgesetz über die Forschung am Menschen (Bundesgesetz über die Forschung am Menschen, HFG) vom 30. September 2011/ Loi fédérale relative à la recherche sur l'être humain (loi relative à la recherche sur l'être humain, LRH) du 30 septembre 2011. (http://www.bag.admin.ch/themen/medizin/00701/00702/07558/index.html?lang=de)
5. Verordnung über klinische Versuche in der Humanforschung (Verordnung über klinische Versuche, KlinV) vom 20. September 2013 / Ordonnance sur les essais cliniques dans le cadre de la recherche sur l'être humain (Ordonnance sur les essais cliniques, OClin) du 20 septembre 2013. (http://www.bag.admin.ch/themen/medizin/00701/00702/12310/index.html?lang=de)
6. Heilmittelgesetz, HMG Bundesgesetz über Arzneimittel und Medizinprodukte (Heilmittelgesetz, HMG) vom 15. Dezember 2000/Loi fédérale sur les médicaments et les dispositifs médicaux (Loi sur les produits thérapeutiques, LPT) du 15 décembre 2000. (http://www.admin.ch/ch/d/sr/8/812.21.de.pdf)
7. ISO 14155:2011 Clinical investigation of medical devices for human subjects -- Good clinical practice (www.iso.org)
8. ISO 10993 Biological evaluation of medical devices (www.iso.org)
9. WHO, International Clinical Trials Registry Platform (ICTRP) (http://www.who.int/ictrp/en/)

Amrhein PC, Miller WR, Yahne CE*, et al.* (2003) Client commitment language during motivational interviewing predicts drug use outcomes. *J Consult Clin Psychol* **71**:862-78.

Apodaca TR and Longabaugh R. (2009) Mechanisms of change in motivational interviewing: a review and preliminary evaluation of the evidence. *Addiction* **104**:705-15.

Babor TF, Caetano R, Casswell S*, et al.* (2010) Alcohol: No Ordinary Commodity. Research and Public Policy. New York: Oxford University Press.

Babor TF, Higgins-Biddle JC, Saunders JB*, et al.* (2001) AUDIT - The Alcohol Use Disorders Identification Test. 2nd ed. Geneva: World Health Organization.

Baer JS, Beadnell B, Garrett SB*, et al.* (2008) Adolescent Change Language Within a Brief Motivational Intervention and Substance Use Outcomes. *Psychology of Addictive Behaviors* **22**:570-5.

Barnett E, Spruijt-Metz D, Moyers TB*, et al.* (2014) Bidirectional Relationships Between Client and Counselor Speech: The Importance of Reframing. *Psychol Addict Behav*.

Bem DJ. (1972) Self-perception theory. In Berkowitz L (Ed.), *Advances in experimental social psychology* (New York: Academic Press, pp. 1-62.

Bertholet N, Adam A, Faouzi M*, et al.* (2014) Admissions of patients with alcohol intoxication in the Emergency Department: a growing phenomenon. *Swiss Med Wkly* **144**:w13982.

Bertholet N, Cunningham JA, Faouzi M*, et al.* (2015) Internet-based brief intervention for young men with unhealthy alcohol use: a randomized controlled trial in a general population sample. *Addiction* **110**:1735-43.

Bien TH, Miller WR, Tonigan JS. (1993) Brief interventions for alcohol problems: a review. *Addiction* **88**:315-35.

Boardman T, Catley D, Grobe JE*, et al.* (2006) Using motivational interviewing with smokers: Do therapist behaviors relate to engagement and therapeutic alliance? *J Subst Abuse Treat* **31**:329-39.

Carey KB, Scott-Sheldon LA, Carey MP*, et al.* (2007) Individual-level interventions to reduce college student drinking: a meta-analytic review. *Addict Behav* **32**:2469-94.

Carey KB, Scott-Sheldon LA, Elliott JC*, et al.* (2012) Face-to-face versus computer-delivered alcohol interventions for college drinkers: a meta-analytic review, 1998 to 2010. *Clinical Psychology Review* **32**:690-703.

Catley D, Harris KJ, Mayo MS*, et al.* (2006) Adherence to principles of Motivational Interviewing and client within-session behavior. *Behavioural and Cognitive Psychotherapy* **34**:43-56.

Cowell AJ, Brown JM, Mills MJ*, et al.* (2012) Cost-effectiveness analysis of motivational interviewing with feedback to reduce drinking among a sample of college students. *J Stud Alcohol Drugs* **73**:226-37.

Creswell JW. (2014) A Concise Introduction to Mixed Methods Research. Thousand Oaks, CA: Sage Publications.

Crunelle CL, Yegles M, De Doncker M*, et al.* (2015) Influence of repeated permanent coloring and bleaching on ethyl glucuronide concentrations in hair from alcohol-dependent patients. *Forensic Sci Int* **247**:18-22.

Crunelle CL, Yegles M, van Nuijs AL*, et al.* (2014) Hair ethyl glucuronide levels as a marker for alcohol use and abuse: a review of the current state of the art. *Drug Alcohol Depend* **134**:1-11.

Daeppen JB, Bertholet N, Gaume J*, et al.* (2011) Efficacy of brief motivational intervention in reducing binge drinking in young men: A randomized controlled trial. *Drug Alcohol Depend* **113**:69-75.

de Boer AG, van Lanschot JJ, Stalmeier PF*, et al.* (2004) Is a single-item visual analogue scale as valid, reliable and responsive as multi-item scales in measuring quality of life? *Qual Life Res* **13**:311-20.

European Monitoring Centre for Drugs and Drug Addiction. (2016) Emergency department based brief interventions for individuals with substance related problems: a review of effectiveness. Luxembourg: Publications Office of the European Union.

Field C, Walters S, Marti CN*, et al.* (2014) A multisite randomized controlled trial of brief intervention to reduce drinking in the trauma care setting: how brief is brief? *Ann Surg* **259**:873-80.

Gaume J, Bertholet N, Faouzi M*, et al.* (2010) Counselor motivational interviewing skills and young adult change talk articulation during brief motivational interventions. *J Subst Abuse Treat* **39**:272-81.

Gaume J, Bertholet N, Faouzi M*, et al.* (2013) Does change talk during brief motivational interventions with young men predict change in alcohol use? *J Subst Abuse Treat* **44**:177-85.

Gaume J, Gmel G, Daeppen JB. (2008) Brief alcohol interventions: do counsellors' and patients' communication characteristics predict change? *Alcohol Alcohol* **43**:62-9.

Gaume J, Gmel G, Faouzi M*, et al.* (2011) Is brief motivational intervention effective in reducing alcohol use among young men voluntarily receiving it? A randomized controlled trial. *Alcohol Clin Exp Res* **35**:1822-30.

Gaume J, Gmel G, Faouzi M*, et al.* (2009) Counselor skill influences outcomes of brief motivational interventions. *J Subst Abuse Treat* **37**:151-9.

Gaume J, Longabaugh R, Magill M*, et al.* (2016) Under What Conditions? Therapist and Client Characteristics Moderate the Role of Change Talk in Brief Motivational Intervention. *J Consult Clin Psychol*.

Gaume J, Magill M, Longabaugh R*, et al.* (*under review*) Influence of counselor characteristics and behaviors on the effectiveness of a brief motivational intervention for heavy drinking in young adults - A randomized controlled trial.

Gaume J, McCambridge J, Bertholet N*, et al.* (2014) Mechanisms of action of brief alcohol interventions remain largely unknown - a narrative review. *Front Psychiatry* **5**:108.

Ham LS, Stewart SH, Norton PJ*, et al.* (2005) Psychometric assessment of the Comprehensive Effects of Alcohol questionnaire: Comparing a brief version to the original full scale. *Journal of Psychopathology and Behavioral Assessment* **27**:141-58.

Hayes AF. (2013) Introduction to mediation, moderation, and conditional process analysis. New York, NY: The Guilford Press.

Horvath AO and Greenberg LS. (1989) Development and validation of the Working Alliance Inventory. *Journal of Counseling Psychology* **36**:223-33.

Imlach FG, Butler S, Cech T*, et al.* (2011) How do intoxicated patients impact staff in the emergency department? An exploratory study. *N Z Med J* **124**:14-23.

Juarez P, Walters ST, Daugherty M*, et al.* (2006) A randomized trial of motivational interviewing and

feedback with heavy drinking college students. *J Drug Educ* **36**:233-46.

Karno MP and Longabaugh R. (2007) Does matching matter? Examining matches and mismatches between patient attributes and therapy techniques in alcoholism treatment. *Addiction* **102**:587-96.

Kerekes I and Yegles M. (2013) Coloring, bleaching, and perming: influence on EtG content in hair. *Ther Drug Monit* **35**:527-9.

Kharbouche H, Faouzi M, Sanchez N*, et al.* (2012) Diagnostic performance of ethyl glucuronide in hair for the investigation of alcohol drinking behavior: a comparison with traditional biomarkers. *Int J Legal Med* **126**:243-50.

Kiluk BD, Dreifuss JA, Weiss RD*, et al.* (2013) The Short Inventory of Problems - revised (SIP-R): psychometric properties within a large, diverse sample of substance use disorder treatment seekers. *Psychol Addict Behav* **27**:307-14.

Kintz P. (2015) 2014 Consensus for the use of alcohol markers in hair for assessment of both abstinence and chronic excessive alcohol consumption. *Forensic Sci Int* **249**:A1-2.

Kroenke K, Spitzer RL, Williams JB*, et al.* (2009) An ultra-brief screening scale for anxiety and depression: the PHQ-4. *Psychosomatics* **50**:613-21.

Lee CS, Baird J, Longabaugh R*, et al.* (2010) Change plan as an active ingredient of brief motivational interventions for reducing negative consequences of drinking in hazardous drinking emergency-department patients. *J Stud Alcohol Drugs* **71**:726-33.

Lee CS, Longabaugh R, Baird J*, et al.* (2007) Do patient intervention ratings predict alcohol-related consequences? *Addict Behav* **32**:3136-41.

Liang K-Y and Zeger SL. (1986) Longitudinal data analysis using generalized linear models. *Biometrika* **73**:13-22.

Little RJ and Rubin DB. (2002) Statistical analysis with missing data. 2nd ed. New York: Wiley & Sons.

Longabaugh R, Donovan DM, Karno MP*, et al.* (2005) Active ingredients: How and why evidence-based alcohol behavioral treatment interventions work. *Alcoholism-Clinical and Experimental Research* **29**:235-47.

Longabaugh R and Magill M. (2011) Recent advances in behavioral addiction treatments: focusing on mechanisms of change. *Curr Psychiatry Rep* **13**:382-9.

Longabaugh R, Minugh PA, Nirenberg TD*, et al.* (1995) Injury as a motivator to reduce drinking. *Acad Emerg Med* **2**:817-25.

Lundahl B and Burke BL. (2009) The effectiveness and applicability of motivational interviewing: a practice-friendly review of four meta-analyses. *Journal of Clinical Psychology* **65**:1232-45.

Magill M, Apodaca TR, Barnett NP*, et al.* (2010) The route to change: Within-session predictors of change plan completion in a motivational interview. *J Subst Abuse Treat* **38**:299-305.

Magill M, Gaume J, Apodaca TR*, et al.* (2014) The Technical Hypothesis of Motivational Interviewing: A Meta-Analysis of MI's Key Causal Model. *J Consult Clin Psychol*.

Maxwell JA. (2005) Qualitative Research Design: An Interactive Approach. Thousand Oaks, CA, USA: Sage.

McCambridge J, McAlaney J, Rowe R. (2011) Adult consequences of late adolescent alcohol consumption: a systematic review of cohort studies. *PLoS Med* **8**:e1000413.

McCambridge J and Rollnick S. (2014) Should brief interventions in primary care address alcohol problems more strongly? *Addiction* **109**:1054-8.

McCormack RP, Gallagher T, Goldfrank LR*, et al.* (2014) Including Frequent Emergency Department Users With Severe Alcohol Use Disorders in Research: Assessing Capacity. *Ann Emerg Med*.

McNally AM, Palfai TP, Kahler CW. (2005) Motivational interventions for heavy drinking college students: examining the role of discrepancy-related psychological processes. *Psychol Addict Behav* **19**:79-87.

Miller WR, Benefield RG, Tonigan JS. (1993) Enhancing Motivation for Change in Problem Drinking - a Controlled Comparison of 2 Therapist Styles. *J Consult Clin Psychol* **61**:455-61.

Miller WR and Moyers TB. (2015) The forest and the trees: relational and specific factors in addiction treatment. *Addiction* **110**:401-13.

Miller WR and Rollnick S. (2013) Motivational Interviewing: Helping People Change. 3rd ed. New York,

NY: Guilford Press.

Miller WR and Rose GS. (2009) Toward a theory of motivational interviewing. *Am Psychol* **64**:527-37.

Miller WR and Wilbourne PL. (2002) Mesa Grande: a methodological analysis of clinical trials of treatments for alcohol use disorders. *Addiction* **97**:265-77.

Monti PM, Colby SM, Barnett NP*, et al.* (1999) Brief intervention for harm reduction with alcohol-positive older adolescents in a hospital emergency department. *J Consult Clin Psychol* **67**:989-94.

Moos RH. (2007) Theory-based active ingredients of effective treatments for substance use disorders. *Drug Alcohol Depend* **88**:109-21.

Moyers TB and Martin T. (2006) Therapist influence on client language during motivational interviewing sessions. *J Subst Abuse Treat* **30**:245-51.

Moyers TB, Martin T, Houck JM*, et al.* (2009) From in-session behaviors to drinking outcomes: a causal chain for motivational interviewing. *J Consult Clin Psychol* **77**:1113-24.

Moyers TB and Miller WR. (2013) Is low therapist empathy toxic? *Psychol Addict Behav* **27**:878-84.

Moyers TB, Miller WR, Hendrickson SM. (2005) How does motivational interviewing work? Therapist interpersonal skill predicts client involvement within motivational interviewing sessions. *J Consult Clin Psychol* **73**:590-8.

Murphy JG, Benson TA, Vuchinich RE*, et al.* (2004) A comparison of personalized feedback for college student drinkers delivered with and without a motivational interview. *J Stud Alcohol* **65**:200-3.

Newton AS, Dong K, Mabood N*, et al.* (2013) Brief emergency department interventions for youth who use alcohol and other drugs: a systematic review. *Pediatr Emerg Care* **29**:673-84.

Nilsen P, Baird J, Mello MJ*, et al.* (2008) A systematic review of emergency care brief alcohol interventions for injury patients. *J Subst Abuse Treat* **35**:184-201.

Norcross JC. (2011) Psychotherapy Relationships that Work: Evidence-Based Responsiveness. New York: Oxford University Press.

O'Donnell A, Anderson P, Newbury-Birch D*, et al.* (2014) The impact of brief alcohol interventions in primary healthcare: a systematic review of reviews. *Alcohol Alcohol* **49**:66-78.

Pirmohamed M, Brown C, Owens L*, et al.* (2000) The burden of alcohol misuse on an inner-city general hospital. *QJM* **93**:291-5.

Rehm J. (2011) The risks associated with alcohol use and alcoholism. *Alcohol Res Health* **34**:135-43.

Rochon J. (1998) Application of GEE procedures for sample size calculations in repeated measures experiments. *Statistics in Medicine* **17**:1643-58.

Rollnick S. (1998) Readiness, importance and confidence: critical conditions of change in treatment. In Miller WR and Heather N (eds.). *Treating Addictive Behaviour* (2nd ed.). New York: Plenum.

Schuckit MA, Anthenelli RM, Bucholz KK*, et al.* (1995) The time course of development of alcohol-related problems in men and women. *J Stud Alcohol* **56**:218-25.

Shen L and Dillard JP. (2005) Psychometric properties of the Hong psychological reactance scale. *J Pers Assess* **85**:74-81.

Smith AJ, Hodgson RJ, Bridgeman K*, et al.* (2003) A randomized controlled trial of a brief intervention after alcohol-related facial injury. *Addiction* **98**:43-52.

Sobell LC and Sobell MB. (1995) Alcohol consumption measures. In Allen JP and Columbus M (eds.). *Assessing alcohol problems: A guide for clinicians and researchers* (Rockville, MD, USA: National Institute on Alcohol Abuse and Alcoholism, pp. 55-73.

Sommers MS, Dyehouse JM, Howe SR*, et al.* (2006) Effectiveness of brief interventions after alcohol-related vehicular injury: A randomized controlled trial. *J Trauma* **61**:523-31; discussion 32-3.

Sommers MS, Lyons MS, Fargo JD*, et al.* (2013) Emergency department-based brief intervention to reduce risky driving and hazardous/harmful drinking in young adults: a randomized controlled trial. *Alcohol Clin Exp Res* **37**:1753-62.

Spirito A, Monti PM, Barnett NP*, et al.* (2004) A randomized clinical trial of a brief motivational intervention for alcohol-positive adolescents treated in an emergency department. *J Pediatr* **145**:396-402.

Taggart IH, Ranney ML, Howland J*, et al.* (2013) A systematic review of emergency department interventions for college drinkers. *J Emerg Med* **45**:962-8.

Tanner-Smith EE and Lipsey MW. (2014) Brief alcohol interventions for adolescents and young adults:

A systematic review and meta-analysis. *J Subst Abuse Treat*.

Tevyaw TO and Monti PM. (2004) Motivational enhancement and other brief interventions for adolescent substance abuse: foundations, applications and evaluations. *Addiction* **99 Suppl 2**:63-75.

Verelst S, Moonen PJ, Desruelles D*, et al.* (2012) Emergency department visits due to alcohol intoxication: characteristics of patients and impact on the emergency room. *Alcohol Alcohol* **47**:433-8.

Walters ST, Vader AM, Harris TR*, et al.* (2009) Dismantling motivational interviewing and feedback for college drinkers: a randomized clinical trial. *J Consult Clin Psychol* **77**:64-73.

Walton MA, Goldstein AL, Chermack ST*, et al.* (2008) Brief alcohol intervention in the emergency department: moderators of effectiveness. *J Stud Alcohol Drugs* **69**:550-60.

Wechsler H, Davenport A, Dowdall G*, et al.* (1994) Health and behavioral consequences of binge drinking in college. A national survey of students at 140 campuses. *JAMA* **272**:1672-7.

WHO. (2011) Global status report on alcohol and health. Geneva: World Health Organization.

Wicki M and Stucki S (2014). Hospitalisierungen aufgrund von Alkohol-Intoxikation oder Alkoholabhängigkeit bei Jugendlichen und Erwachsenen - Eine Analyse der Schweizerischen "Medizinischen Statistik der Krankenhäuser“ 2003 bis 2012. Lausanne, Switzerland: Addiction switzerland. Available at http://www.addictionsuisse.ch/fileadmin/user_upload/DocUpload/RR_73.

Wicki M, Wurdak M, Kuntsche E (2014). Effektivität psychosozialer Interventionen im Spital bei Alkohol-Intoxikation: Eine systematische Literaturübersicht [Effectiveness of psychosocial interventions in the hospital for alcohol intoxication: a systematic literature review]. Lausanne: Addiction Switzerland. Available at http://www.addictionsuisse.ch/fileadmin/user_upload/DocUpload/SUCHT-SCHWEIZ-Effektivitaet-psychosozialer-Interventionen-im_Spital-bei-Alkohol-Intoxikation-FB69.pdf.

17. APPENDICES

- Case Report Form (CRF)
- Patient Information and informed consent forms

These forms were uploaded on the BASEC website.
