# Supplementary material for: Effect of a Novel Brief Motivational Intervention for Alcohol-Intoxicated Young Adults in the Emergency Department: A Randomized Clinical Trial
Source: JAMA Netw Open. 2022 Oct 21;5(10):e2237563. doi: 10.1001/jamanetworkopen.2022.37563 (PMC9587483; doi:10.1001/jamanetworkopen.2022.37563)
Supplement: Supplement 2. — eMethods. Supplemental Methods eReferences eTable 1. Intervention Fidelity eTable 2. Sensitivity Analyses eTable 3. Other Countries of Citizenship [file jamanetwopen-e2237563-s002.pdf]

Supplemental Online Content

Gaume J, Bertholet N, McCambridge J, et al. Effect of a novel brief motivational intervention for alcohol-intoxicated young adults in the emergency department: a randomized clinical trial. *JAMA Netw Open.* 2022;5(10):e2237563. doi:10.1001/jamanetworkopen.2022.37563

eMethods. Supplemental Methods

eReferences

eTable 1. Intervention Fidelity

eTable 2. Sensitivity Analyses

eTable 3. Other Countries of Citizenship

This supplemental material has been provided by the authors to give readers additional information about their work.

© 2022 Gaume J et al. *JAMA Network Open.*

eMethods. Supplemental Methods

Ethyl glucuronide in head hair sample

Participants were asked to provide a head hair sample to measure Ethyl glucuronide (EtG), the only biomarker that can discriminate chronic heavy use from social or no use. [1] We used the cutoff value of >30 pg/mg of hair proposed by the Society of Hair Testing [2] to detect chronic heavy use (sensitivity: 96%, specificity: 99%). [1]

Participants were asked for consent to take a 2-3 centimeters long strand of hair (diameter of pencil lead) at baseline, 6-, and 12-month follow-up. Hair sampling was performed at the end of inclusion procedures at baseline and during a specific appointment with a research assistant at Lausanne University Hospital at 6- and 12-month follow-up. A CHF 20.- incentive was offered to compensate for each hair sampling. Travel expenses to Lausanne University Hospital were reimbursed at 6- and 12-month follow-up.

At baseline, N=124 (36.1%) provided a sample, N=90 (26.2%) refused, and N=129 (37.6%) had hair too short (<2cm). At 6-month, N=123 (45.9%) provided a sample, N=104 (38.8%) refused, N=29 (10.8%) had hair too short, and N=12 (4.5%) set an appointment but did not show up. At 12-month, N=104 (38.2%) provided a sample, N=91 (33.5%) refused, N=25 (9.2%) had hair too short, N=11 (4.0%) set an appointment but did not show up; in addition hair sampling was cancelled for N=41 (15.1%) due to Covid-19 pandemic measures (ban of all non-essential visits to the hospital from March 15, 2020).

Treatment fidelity

Intervention fidelity was measured using psycholinguistic coding of audio-recorded sessions. There were audio recordings available for N=290 (84.3%). One session was not conducted (early drop out, participant not feeling well). Consent was granted for audio-recording of the session and N=48 (13.9%) refused recording. There were technical problems for the remaining N=5 (1.5%). There were no significant differences between recorded and non-recorded sessions on baseline variables reported in Table 1, except for gender (91.7% recorded for female vs. 81.9% for male, Chi2 (1) = 4.55, P = 0.03).

Recorded sessions were coded using the validated Motivational Interviewing Skill Code (MISC), version 2.5. [3,4] Global ratings, which characterize the overall interaction, were assessed on a 5-point Likert scale, with the coder assuming a neutral beginning score of “3”. Global ratings comprised the clinician’s level of

© 2022 Gaume J et al. *JAMA Network Open.*

acceptance, empathy, autonomy support, collaboration, and evocation, and patient's level of self-exploration. In addition, we also evaluated clinician's skills to cultivate change talk and soften sustain talk, based on similar scales available in the validated Motivational Interviewing Treatment Integrity, version 4.2. [5]

Then, each clinician's and patient's utterance were categorized using one of the 19 counselor and 8 client MISC codes. Several summary scores of counselor behaviors can be derived as indicators of the quality of MI. [6] In the present report, we used:

- the percentage of MI-consistent behaviors (i.e. total MI-consistent/total therapist's behaviors*100; MI-consistent behaviors being advise with permission, affirm, emphasize control, open question, simple and complex reflections, reframe, and support)
- the percentage of open questions (total open questions/total open and closed questions*100)
- the percentage of complex reflections (total complex reflections /total simple and complex reflections*100)
- the ratio of reflections over questions (total reflections/total questions)
- the patient's percentage of change talk (total change talk utterances/total patient's utterances*100).

We created two additional scores related to giving information and giving advice (which were expected to be higher in the brief advice condition):

- the percentage of giving information (frequency of giving information/total therapist's behaviors*100)
- the percentage of advice without permission (frequency of advice without permission/total advices with or without permission*100).

A random sample of 58 sessions (20%) was double coded to assess inter-rater reliability while coders were blinded whether another was also coding the session. Intra-class correlations (ICC) was computed using two-way mixed-effect models specifying consistency of agreement and individual measurements for each code to account for inter-rater reliability. According to the categorization of Cicchetti [7], agreement between coders was good to excellent for retained measures (ICC ranging between 0.70 and 0.98 for global ratings and 0.82 and 0.96 for behavior counts), except for the percentage of complex reflections and the percentage of advice without permission for which ICC was fair (0.51 and 0.44, respectively).

Intervention length, global ratings and behavior counts were compared across treatment groups using two-sample Wilcoxon rank-sum test since data were not normally distributed.

eReferences

1. Crunelle CL, Yegles M, van Nuijs AL, et al. Hair ethyl glucuronide levels as a marker for alcohol use and abuse: a review of the current state of the art. *Drug Alcohol Depend.* 2014;134:1-11.
2. Kintz P. 2014 Consensus for the use of alcohol markers in hair for assessment of both abstinence and chronic excessive alcohol consumption. *Forensic Sci Int.* 2015;249:A1-2.
3. Moyers T, Martin T, Catley D, Harris K, Ahluwalia JS. Assessing the integrity of motivational interviewing interventions: Reliability of the motivational interviewing skills code. *Behav Cogn Psychother.* 2003;31:177-184.
4. Houck JM, Moyers TB, Miller WR, Glynn LH, Hallgren KA. Motivational Interviewing Skill Code (MISC) 2.5. Albuquerque, NM, USA: University of New Mexico, Center on Alcoholism, Substance Abuse, and Addictions; 2014. http://casaa.unm.edu/download/misc25.pdf.
5. Moyers TB, Rowell LN, Manuel JK, Ernst D, Houck JM. The Motivational Interviewing Treatment Integrity Code (MITI 4): Rationale, Preliminary Reliability and Validity. *J Subst Abuse Treat.* 2016;65:36-42.
6. Miller WR, Moyers TB, Ernst D, Amrhein PC. Manual for the Motivational Interviewing Skill Code (MISC). Albuquerque, NM: University of New Mexico; 2008. http://casaa.unm.edu/download/misc.pdf.
7. Cicchetti DV. Guidelines, criteria, and rules of thumb for evaluating normed and standardized assessment instruments in psychology. *Psychological Assessment.* 1994;6(4):284-290.

© 2022 Gaume J et al. *JAMA Network Open.*

eTable 1. Intervention fidelity

		BA			bMI		Test	
	25th pct	Median	75th pct	25th pct	Median	75th pct	z	*P*
Session length (minutes) [a]								
Baseline intervention	2	3	4	30	37	45	-16.09	<.0001
1-week booster	n/a	n/a	n/a	8	11	16	n/a	n/a
1-month booster	n/a	n/a	n/a	8	11	19	n/a	n/a
3-month booster	n/a	n/a	n/a	8	11	17	n/a	n/a
Global ratings (1-5 scale) [b]								
Acceptance	3	3	3	4	5	5	-14.75	<.0001
Empathy	1	1	1	4	5	5	-15.77	<.0001
Autonomy support	3	3	3	4	5	5	-14.56	<.0001
Collaboration	1	1	1	4	5	5	-15.80	<.0001
Evocation	1	1	1	4	4	5	-15.41	<.0001
Cultivate change talk	1	1	1	3	4	5	-15.24	<.0001
Soften sustain talk	3	3	3	4	4	4	-12.95	<.0001
Self-exploration	1	1	1	3	4	4	-14.94	<.0001
Behavior counts [b]								
% MI-consistent	0	0	14.3	55.2	62.3	72.2	-14.78	<.0001
% Open question	0	0	0	41.2	50	60.2	-15.20	<.0001
% Complex reflection	0	0	0	24.1	34.1	44.2	-14.78	<.0001
Ratio reflections/questions	0	1	1	0.8	1.3	1.9	-5.32	<.0001
% Change talk	0	0	0	13.7	19.4	27.9	-14.28	<.0001
% Giving information	50	57.1	71.4	1.4	4.4	9.0	14.68	<.0001
% Advice without permission	0	100	100	0	0	0	7.82	<.0001

Abbreviations: BA, Brief Advice; bMI, Brief Motivational Intervention; pct, percentile; n/a, not applicable (i.e., no booster session of BA).

[a] Session length was entered for all interventions (N=343).

[b] Global ratings and behaviors counts measured among N=290 coded sessions (140 bMI and 150 BA).

© 2022 Gaume J et al. *JAMA Network Open.*

eTable 2. Sensitivity analyses

	1) Adjusted for age and sex					2) Robust standard errors					3) Multiple imputation				
	Coef.	*SE*	*P*	*[95%*	*CI]*	*Coef.*	*SE*	*P*	*[95%*	*CI]*	*Coef.*	*SE*	*P*	*[95%*	*CI]*
Heavy drinking days [a]															
bMI (vs. BA)	0.12	0.11	0.25	-0.09	0.34	0.09	0.12	0.46	-0.15	0.32	0.09	0.10	0.40	-0.11	0.28
Time (months) [b]	0.04	0.01	<0.001	0.02	0.06	0.04	0.01	0.001	0.02	0.06	0.04	0.01	<0.001	0.02	0.06
bMI X Time	-0.03	0.01	0.03	-0.05	0.00	-0.03	0.01	0.047	-0.06	0.00	-0.03	0.01	0.02	-0.05	0.00
Short Inventory of Problems [a]															
bMI (vs. BA)	0.05	0.12	0.64	-0.18	0.29	0.06	0.10	0.56	-0.14	0.25	0.05	0.11	0.68	-0.17	0.26
Time (months) [b]	-0.01	0.01	0.20	-0.03	0.01	-0.01	0.01	0.23	-0.03	0.01	-0.01	0.01	0.29	-0.03	0.01
bMI X Time	-0.01	0.01	0.73	-0.03	0.02	-0.01	0.01	0.70	-0.03	0.02	0.00	0.02	0.83	-0.04	0.03
Weekly drinking amount [a]															
bMI (vs. BA)	0.09	0.09	0.33	-0.09	0.27	0.09	0.10	0.37	-0.10	0.28	0.09	0.08	0.31	-0.08	0.25
Time (months) [b]	0.03	0.01	0.003	0.01	0.04	0.03	0.01	0.01	0.01	0.05	0.02	0.01	0.004	0.01	0.04
bMI X Time	-0.01	0.01	0.35	-0.04	0.01	-0.01	0.01	0.42	-0.04	0.02	-0.01	0.01	0.66	-0.03	0.02
Consequences [a]															
bMI (vs. BA)	0.02	0.17	0.92	-0.31	0.35	0.01	0.15	0.94	-0.29	0.31	0.01	0.16	0.95	-0.31	0.33
Time (months) [b]	-0.02	0.02	0.30	-0.06	0.02	-0.02	0.02	0.28	-0.06	0.02	-0.02	0.02	0.38	-0.05	0.02
bMI X Time	0.03	0.03	0.25	-0.02	0.09	0.03	0.03	0.25	-0.02	0.09	0.03	0.03	0.29	-0.03	0.09

© 2022 Gaume J et al. *JAMA Network Open.*

	1) Adjusted for age and sex					2) Robust standard errors					3) Multiple imputation				
	Coef.	*SE*	*P*	*[95%*	*CI]*	*Coef.*	*SE*	*P*	*[95%*	*CI]*	*Coef.*	*SE*	*P*	*[95%*	*CI]*
Hazardous alcohol use [c]															
bMI (vs. BA)	0.12	0.27	0.65	-0.41	0.66	N/A					0.07	0.27	0.81	-0.47	0.60
Readmission in the ED (self-reported) [d]															
bMI (vs. BA)	0.19	0.29	0.51	-0.38	0.76	0.20	0.29	0.49	-0.36	0.76	0.23	0.29	0.43	-0.35	0.81
Time (months) [b]	0.05	0.04	0.24	-0.03	0.12	0.05	0.04	0.23	-0.03	0.12	0.04	0.04	0.25	-0.03	0.12
bMI X Time	-0.08	0.06	0.15	-0.19	0.03	-0.08	0.05	0.14	-0.19	0.03	-0.08	0.06	0.18	-0.19	0.04
Started alcohol treatment (self-reported) [d]															
bMI (vs. BA)	1.04	0.46	0.03	0.13	1.94	0.85	0.52	0.10	-0.17	1.86	0.90	0.44	0.04	0.04	1.76
Time (months) [b]	0.04	0.07	0.60	-0.11	0.19	0.03	0.08	0.66	-0.12	0.19	0.03	0.07	0.68	-0.12	0.18
bMI X Time	-0.11	0.10	0.27	-0.29	0.08	-0.11	0.10	0.31	-0.31	0.10	-0.10	0.09	0.28	-0.28	0.08
Readmission in the ED (medical record) [c]															
bMI (vs. BA)	0.27	0.33	0.41	-0.37	0.91	N/A					0.26	0.32	0.42	-0.38	0.90

© 2022 Gaume J et al. *JAMA Network Open.*

	**1) Adjusted for age and sex**					**2) Robust standard errors**					**3) Multiple imputation**				
	Coef.	*SE*	*P*	*[95%*	*CI]*	*Coef.*	*SE*	*P*	*[95%*	*CI]*	*Coef.*	*SE*	*P*	*[95%*	*CI]*
Started alcohol treatment (medical record) [c]															
bMI (vs. BA)	1.27	0.59	0.03	0.12	2.42	N/A					1.26	0.62	0.04	0.05	2.47
EtG indicating heavy use [e]															
bMI (vs. BA)	-0.29	0.29	0.33	-0.86	0.29	-0.33	0.29	0.26	-0.90	0.24	-0.43	0.30	0.15	-1.01	0.16
Time (months) [b]	-0.09	0.04	0.01	-0.16	-0.02	-0.06	0.03	0.06	-0.13	0.00	-0.04	0.03	0.13	-0.09	0.01
bMI X Time	0.02	0.05	0.63	-0.07	0.12	0.02	0.05	0.70	-0.07	0.11	-0.01	0.04	0.89	-0.09	0.07

Abbreviations: Coef., regression coefficients; SE, Standard Error; CI, Confidence Interval; bMI, Brief Motivational Intervention; BA, Brief Advice; ED, Emergency Department; EtG, Ethyl Glucuronide.

[a] Generalized estimating equation model with negative binomial distribution, log link, and exchangeable correlation structure; adjusted for a corresponding baseline measure (see Methods).

[b] Follow-up months were mean-centered.

[c] Logistic regression model; adjusted for a corresponding baseline measure (see Methods).

[d] Generalized estimating equation model with binomial distribution, logit link, and exchangeable correlation structure; adjusted for a corresponding baseline measure (see Methods).

[e] Generalized estimating equation model with binomial distribution, logit link, and exchangeable correlation structure.

© 2022 Gaume J et al. *JAMA Network Open.*

eTable 3. Other Countries of Citizenship

Other country of citizenship	All	BA	Brief MI
France	47	20	27
Portugal	18	10	8
Spain	7	6	1
Ecuador	5	3	2
Italia	5	2	3
Morocco	5	2	3
Democratic Republic of Congo	3	0	3
United Kingdom	3	2	1
Belgium	2	2	0
Chile	2	1	1
Germany	2	2	0
Kosovo	2	1	1
Macedonia	2	1	1
Russia	2	1	1
Algeria	1	1	0

© 2022 Gaume J et al. *JAMA Network Open.*

Brazil	1	1	0
Cameroun	1	0	1
Canada	1	1	0
Colombia	1	1	0
Eritrea	1	1	0
India	1	1	0
Ireland	1	1	0
Mongolia	1	0	1
Nepal	1	1	0
Norway	1	1	0
Poland	1	0	1
Somalia	1	0	1

© 2022 Gaume J et al. *JAMA Network Open.*

© 2022 Gaume J et al. *JAMA Network Open.*
