# Supplementary material for: Effect of a Novel Brief Motivational Intervention for Alcohol-Intoxicated Young Adults in the Emergency Department: A Randomized Clinical Trial
Source: JAMA Netw Open. 2022 Oct 21;5(10):e2237563. doi: 10.1001/jamanetworkopen.2022.37563 (PMC9587483; doi:10.1001/jamanetworkopen.2022.37563)
Supplement: Supplement 3. — Data Sharing Statement [file jamanetwopen-e2237563-s003.pdf]

Data Sharing Statement

Gaume. Effect of a Novel Brief Motivational Intervention for Alcohol-Intoxicated Young Adults in the Emergency Department. *JAMA Netw Open*. Published October 21, 2022. doi:10.1001/jamanetworkopen.2022.37563

Data
Data available: Yes
Data types: Deidentified participant data, Data dictionary
How to access data: A dataset comprising the variables used in the present analysis will be available open access, together with a codebook file (variables dictionary). These data will be stored on the Zenodo repository (https://zenodo.org/) when the article is published and a DOI link to the dataset page will be provided. As expected by the Swiss National Science Foundation, the data will be available for everyone to reuse without restriction. Based on ethical regulations, only deidentified data are available.
When available: With publication

Supporting Documents
Document types: None

Additional Information
Who can access the data: As expected by the Swiss National Science Foundation, the data will be available for everyone to reuse without restriction.
Types of analyses: for any purpose
Mechanisms of data availability: without investigator support
